# Regulation of terpenoid biosynthesis by miRNA in *Persicaria minor* induced by *Fusarium oxysporum*

**DOI:** 10.1186/s12864-019-5954-0

**Published:** 2019-07-16

**Authors:** Abdul Fatah A. Samad, Reyhaneh Rahnamaie-Tajadod, Muhammad Sajad, Jaeyres Jani, Abdul Munir Abdul Murad, Normah Mohd Noor, Ismanizan Ismail

**Affiliations:** 1 School of Biosciences and Biotechnology, Faculty of Science and Technology, Universiti Kebangsaan Malaysia, UKM, 43600 Bangi, Selangor Malaysia; 2 Institute of Systems Biology, Universiti Kebangsaan Malaysia, UKM, 43600 Bangi, Selangor Malaysia; 3 Borneo Medical and Health Research Centre, Faculty of Medicine and Health Sciences, Universiti Malaysia Sabah, Kota Kinabalu, Malaysia; 4 Department of Plant Breeding and Genetics, University College of Agriculture & Environmental Sciences, The Islamia University of Bahawalpur, Punjab, Pakistan; 5 Department of Biosciences, Faculty of Science, Universiti Teknologi Malaysia, 81310 Skudai, Johor Malaysia

**Keywords:** Terpenoid biosynthesis, miRNA, *P. minor*, *F. Oxysporum*, Deep sequencing, Post-transcriptional regulation

## Abstract

**Background:**

*Persicaria minor* (kesum) is an herbaceous plant with a high level of secondary metabolite compounds, particularly terpenoids. These terpenoid compounds have well-established roles in the pharmaceutical and food industries. Although the terpenoids of *P. minor* have been studied thoroughly, the involvement of microRNA (miRNA) in terpenoid regulation remains poorly understood and needs to be explored. In this study, *P. minor* plants were inoculated with the pathogenic fungus *Fusarium oxysporum* for terpenoid induction.

**Result:**

SPME GC-MS analysis showed the highest terpenoid accumulation on the 6th day post-inoculation (dpi) compared to the other treatment time points (0 dpi, 3 dpi, and 9 dpi). Among the increased terpenoid compounds, α-cedrene, valencene and β-bisabolene were prominent. *P. minor* inoculated for 6 days was selected for miRNA library construction using next generation sequencing. Differential gene expression analysis showed that 58 miRNAs belonging to 30 families had significantly altered regulation. Among these 58 differentially expressed genes (DEGs), 33 miRNAs were upregulated, whereas 25 miRNAs were downregulated. Two putative novel pre-miRNAs were identified and validated through reverse transcriptase PCR. Prediction of target transcripts potentially involved in the mevalonate pathway (MVA) was carried out by psRobot software, resulting in four miRNAs: pmi-miR530, pmi-miR6173, pmi-miR6300 and a novel miRNA, pmi-Nov_13. In addition, two miRNAs, miR396a and miR398f/g, were predicted to have their target transcripts in the non-mevalonate pathway (MEP). In addition, a novel miRNA, pmi-Nov_12, was identified to have a target gene involved in green leaf volatile (GLV) biosynthesis. RT-qPCR analysis showed that pmi-miR6173, pmi-miR6300 and pmi-nov_13 were downregulated, while miR396a and miR398f/g were upregulated. Pmi-miR530 showed upregulation at 9 dpi, and dynamic expression was observed for pmi-nov_12. Pmi-6300 and pmi-miR396a cleavage sites were detected through degradome sequence analysis. Furthermore, the relationship between miRNA metabolites and mRNA metabolites was validated using correlation analysis.

**Conclusion:**

Our findings suggest that six studied miRNAs post-transcriptionally regulate terpenoid biosynthesis in *P. minor*. This regulatory behaviour of miRNAs has potential as a genetic tool to regulate terpenoid biosynthesis in *P. minor*.

**Electronic supplementary material:**

The online version of this article (10.1186/s12864-019-5954-0) contains supplementary material, which is available to authorized users.

## Background

Humans exploit the secondary metabolites from medicinal plants in the form of flavouring agents, perfumes, insecticides, dyes and drugs, among other products. Previous studies have shown that more than 100,000 phytochemicals have been isolated from different plant sources [1]. According to the British Nutrition Foundation, secondary metabolites (SMs) are divided into four major classes: terpenoids (volatile compounds, carotenoids and glycosides), phenolic compounds (phenolic acids, tannins and flavonoids), nitrogen-containing compounds (cyanogenic glucosides and alkaloids) and sulfur-containing compounds (thionine, defensin and lectin) [2]. The terpenoid classes consist of volatile and non-volatile compounds. Though these compounds exist in complex structures, they all are made up of isoprene units (C5). Most volatile terpenoids are monoterpenes (C10) and sesquiterpenes (C15), while non-volatile terpenoids include diterpenes (C20), triterpenes (C30) and tetraterpenes (C40) [3]. Terpenoids are widely used in pharmaceuticals, cosmetic fragrances and the food industry [4]. To date, more than 40,000 known terpenoid compounds have been identified, mostly from plants [5].

*Persicaria minor* (locally known as kesum) is an aromatic plant that is widespread in Southeast Asia, especially in Malaysia, Thailand, Laos, Indonesia and Vietnam. This plant has been used in dishes and medical purposes for many years. The Malaysian government enlisted *P. minor* in the National Agro Food Policy to ensure its adequate supply and as a platform to strengthen the agricultural-based economy [6]. *P. minor* has also gained attention due to its abundance of secondary metabolites, especially flavonoids and terpenoids, which are used in the food, fragrance and pharmaceutical industries [7]. *P. minor* leaves produce the highest content of terpenoids compared to other organs [8]. These compounds play important roles in attracting pollinators, plant defence, and interactions with unfavourable environments. Two different pathways of terpenoid biosynthesis have been identified: the mevalonate (MVA) and non-mevalonate, or methylerythritol 4-phosphate (MEP), pathways [9]. These pathways operate in the cytoplasm and plastid, respectively [9].

Past achievements in unlocking the genes and enzymes involved in each of these pathways has opened up new possibilities for the assessment of terpenoid biosynthesis [10]. At the molecular level, molecules such as DNA, mRNA, transcription factors, and non-coding RNA play roles in regulating the genes involved in these pathways. In addition, these molecules may interact with each other, which can affect the target gene, thus influencing the production of the secondary metabolite [11]. However, the level of secondary metabolites in plants is relatively low because their production depends on plant species, environmental factors, and nutritional sources [12, 13]. In addition, the type of secondary metabolites produced by the plant is stimulus-dependent. For example, in the majority of cases, terpenoid compounds are produced in high abundance under biotic stress compared to abiotic [14]. Hence, the elicitation technique was introduced to enhance secondary metabolite production in plants using a biotic elicitor [13, 15]. Elicitation using fungi, especially *F. oxysporum*, has been reported to enhance terpenoid contents in plants [16–19].

To understand the changes at the molecular level, the relationship between miRNA and mRNA needs to be explored, because the interaction between these two RNA species may play a significant role in plant secondary metabolite production. miRNAs are a group of non-coding RNAs with small sizes (~ 20 bp) that act as a gene regulators by negatively regulating mRNAs via cleavage or translational inhibition [11, 20]. The first miRNA, lin-4 miRNA, was discovered in *C. elegans* through forward genetic analysis. Later, several methods were developed to discover these tiny RNAs, such as cloning and computational methods [20]. However, these methods had their own limitations. Due to the ability of several miRNAs to target single mRNAs, using forward genetic techniques for miRNA discovery is very difficult unless the miRNA of interest has only one target [11, 20]. The cloning method is also limited to highly expressed miRNAs [20]. Computational approaches could be useful for miRNA discovery but have limitations in detecting novel miRNAs in other species, since these approaches are based on homology searches. In addition, computational approaches still require experimental validation [20]. Next generation sequencing (NGS), a more recent approach, has also been introduced for miRNA study, and this method has been found to be more advanced and efficient. This approach can detect low copy number miRNAs at levels of one transcript per million [21, 22]. To date, a total of 38,589 miRNAs from animals, plants, and viruses have been discovered and deposited in public miRNA databases [23]. These miRNAs have the ability to fine-tune many biological and physiological processes such as plant development and stress response by regulating a number of target genes in a time-sensitive manner [11].

Interestingly, miRNAs were reported to be involved in plant secondary metabolite regulation, such as terpenoid, phenolic and nitrogen-containing compound biosynthesis [1]. These interactions could hold potential for future genetic manipulation. In addition, using miRNA as a tool for genetic engineering could be highly beneficial because single miRNAs can target more than single mRNAs and vice versa. Authenticated in silico miRNA discovery and wet lab approaches for miRNA target validation are required prior to their application to plant genetic systems. Thus, in this study, the regulatory roles of miRNAs in the terpenoid biosynthesis pathway were explored in *P. minor* induced by *F. oxysporum*. Metabolite profiling was carried out for the *F. oxysporum-*inoculated plants to determine the changes in the accumulation of terpenoid compounds. In addition, small RNA libraries were constructed, which led to the characterization and analysis of miRNAs involved in the terpenoid biosynthesis pathway.

## Results and discussion

### Testing of *F. oxysporum* inoculation and terpenoid profiling in *P. minor* by SPME-GCMS

**Fig. 1:**
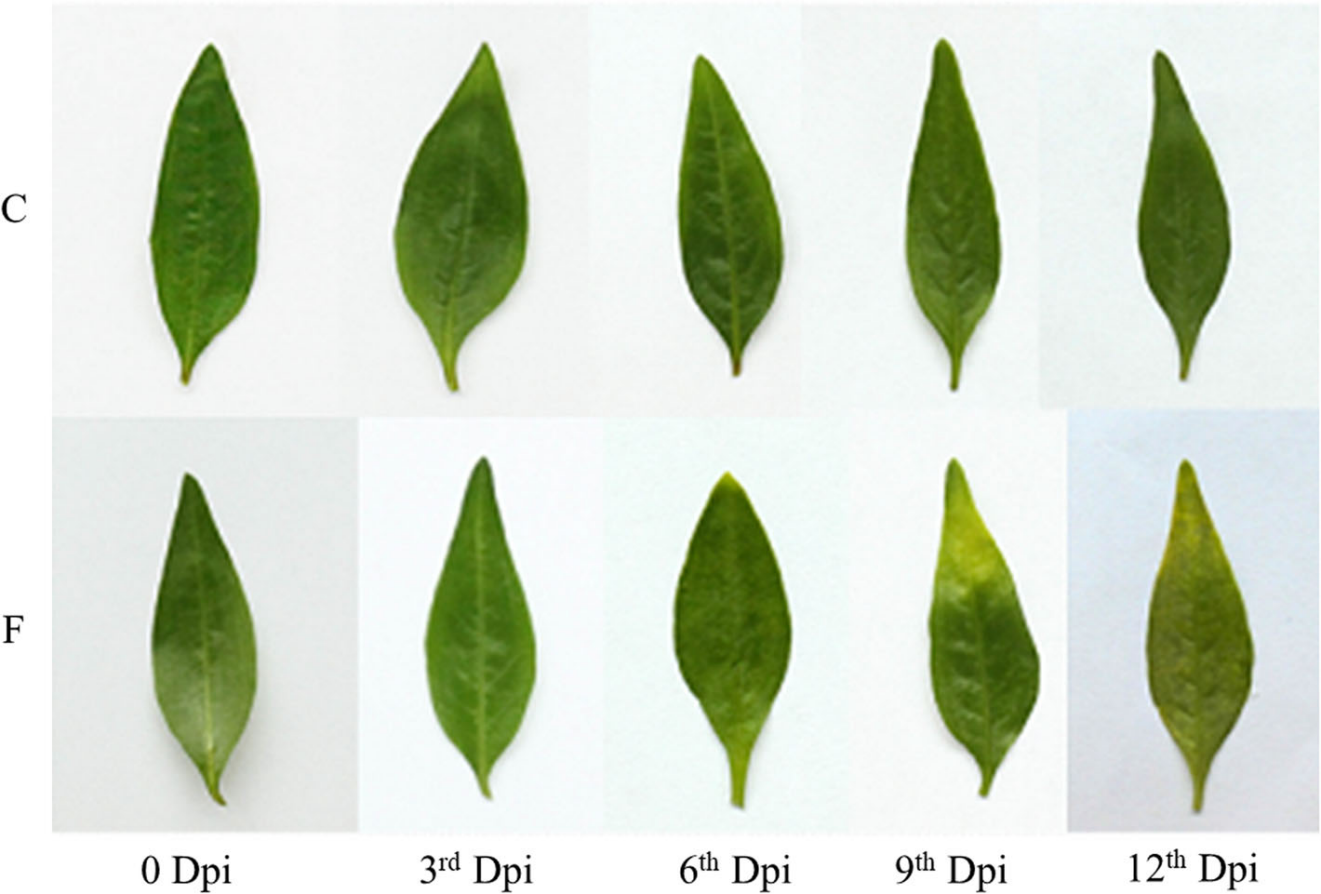
Morphological changes in *P. minor* leaves inoculated with *F. oxysporum* in a time series (0 dpi, 3 dpi, 6 dpi, 9 dpi and 12 dpi)

**Fig. 2:**
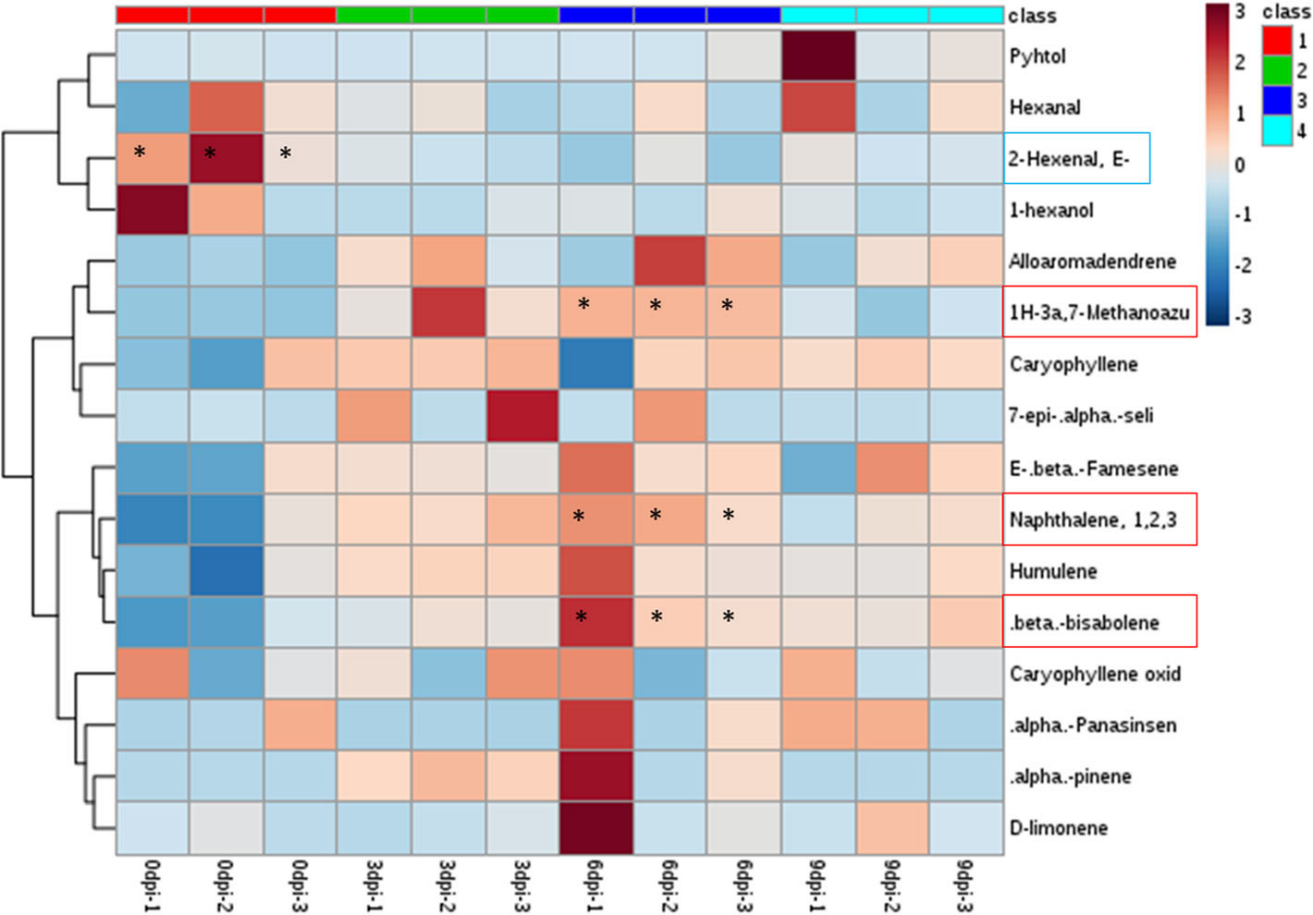
Heat map representing changes in relative metabolite contents of *Fusarium*-inoculated and control plants detected by SPME GC-MS experiments. Asterisks indicate statistically significant differences (*P* < 0.05) among all time points by ANOVA and Fisher’s LSD test. The compounds in the blue and red boxes represent the statistically significant GLV and terpenoids, respectively

To the best of our knowledge, no work on *F. oxysporum* inoculation to induce terpenoid contents has been reported to date in *P. minor*. Therefore, we carried out an inoculation test to determine the elicitation ability of this fungus to increase terpenoid content in *P. minor*. Comparison between mock-inoculated (C) and *Fusarium*-treated (F) plants showed different leaf morphologies over the time point of the treatment (Fig. 1). From the beginning of the treatment (0 dpi) to 3 dpi, there were no morphological changes observed in either of the C or F samples. At 6 dpi, no change was observed in the C samples, while the F samples showed wilting symptoms beginning at the tip of the leaf. At 9 dpi, the wilting spread to the middle of the leaves in the F samples. At 12 dpi, the wilting spread to the whole leaf, which led to the death of the plant. In comparison, no changes were observed in the C samples until the end point, 12 dpi. A previous report mentioned an accumulation of terpenoids when plants were attacked by pathogens [24]. Based on Fig. 1, the first symptom appeared at 6 dpi. Theoretically, the test suggested the highest terpenoid content at 6 dpi in *P. minor* inoculated with *F. oxysporum.* However, the SPME-GCMS result was carried out to reveal the terpenoid content at each time point. The initial SPME-GCMS data are provided in Additional file 1. The heatmap generated (Fig. 2) indicated a significant increase in three volatile compounds: α-cedrene (1H-3a,7-methanoazulene, 2,3,4,7,8,8a-hexahydro-3,6,8,8-tetramethyl-, [3R-(3.α.,3a.beta.,7.beta.,8a.α.)]-), valencene (naphthalene, 1,2,3,5,6,7,8,8a-octahydro-1,8a-dimethyl-7-(1-methylethenyl)-, [1S-(1.α.,7.α.,8a.α.)]-) and β-bisabolene on 6 dpi. Hence, samples at the 6 dpi time point were selected for small RNA library construction.

### High-throughput small RNA sequencing analysis

**Fig. 3:**
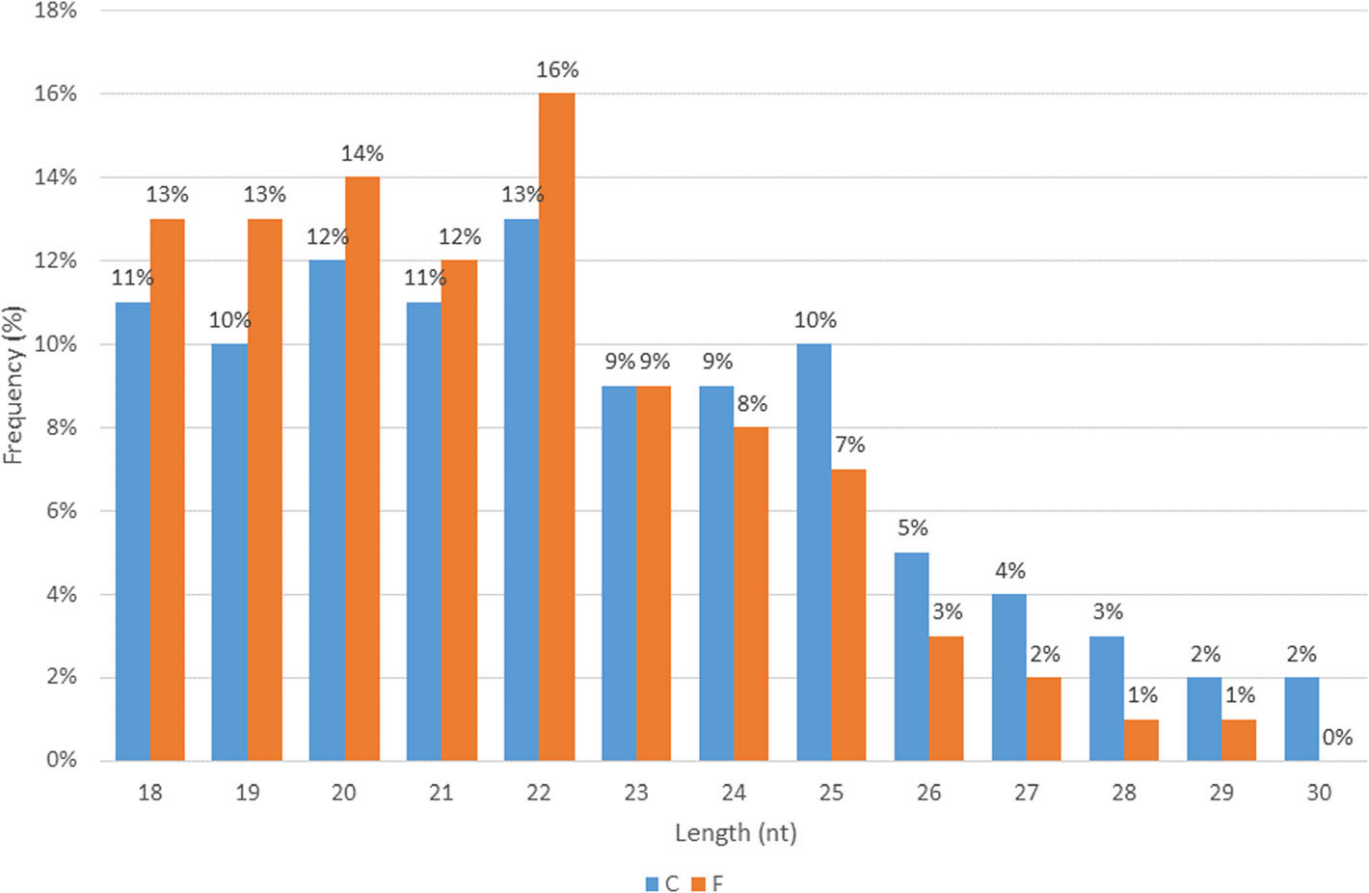
Length distributions of small RNAs in the C and F libraries

Small RNA sequencing for the C and F libraries generated approximately 12,409,685 and 42,985,084 reads, respectively. The average numbers and proportions of the different categories are presented in Table 1. Trimming of adaptor index sequences was carried out, and low-quality reads were removed to produce clean reads. Reads with lengths between 18 nt and 30 nt were used for annotation, while the rest were discarded. In total, approximately 7,724,932 and 25,726,524 unique reads were produced for the C and F libraries, respectively. The length distribution of the small RNAs in the C and F libraries is shown in Fig. 3. The most abundant small RNAs were 22 nt in length, followed by 20 nt, which belonged to the F library. Previous studies have reported that more than 60% of all plant miRNAs are 21 nt in length [25]. The clean reads from each *P. minor* library were annotated according to the miRBase version 21 and Rfam databases. From the total clean reads, the C library generated 111,235 (1.44%) miRNA sequences, which represented 2,610 (0.18%) unique reads. On the other hand, the F library generated 711,498 (2.77%) miRNA sequences, representing 6,131 (0.18%) unique reads. Mapping with Rfam resulted in 15.48 and 18.13% sequences in the C and F libraries, respectively, belonging to non-coding RNAs other than miRNA. The rest of the sequences were marked as unannotated sequences in both libraries.

**Table 1:** Average statistic for deep sequencing results in C and F sample

	Total reads	Percentages (%)	Unique reads	Percentages (%)
C				
Raw reads	12 409 685 ± 7 070 024			
Clean reads	7 724 932 ± 4 736 271	100.00	1 451 635 ± 830 002	100.00
miRNA	111 235 ± 84 067	1.44	2610 ± 1151	0.18
rRNA/tRNA/snoRNA	1 195 917 ± 667 460	15.48	100 356 ± 35 621	6.91
Unannotated	6 417 780 ± 3 984 743	83.07	1 348 660 ± 793 229	92.91
F				
Raw reads	42 985 084 ± 144 059			
Clean reads	25 726 524 ± 15 406396	100.00	3 371 142 ± 1 880 202	100.00
miRNA	711 498 ± 657 406	2.77	6131 ± 2710	0.18
rRNA/tRNA/snoRNA	4 664 038 ± 2 660 762	18.13	182 761 ± 83 483	5.42
Unannotated	20 351 078 ± 12 088 100	79.11	3 182 248 ± 1 794 008	94.40

### Differential expression of miRNAs in C and F libraries

**Fig. 4:**
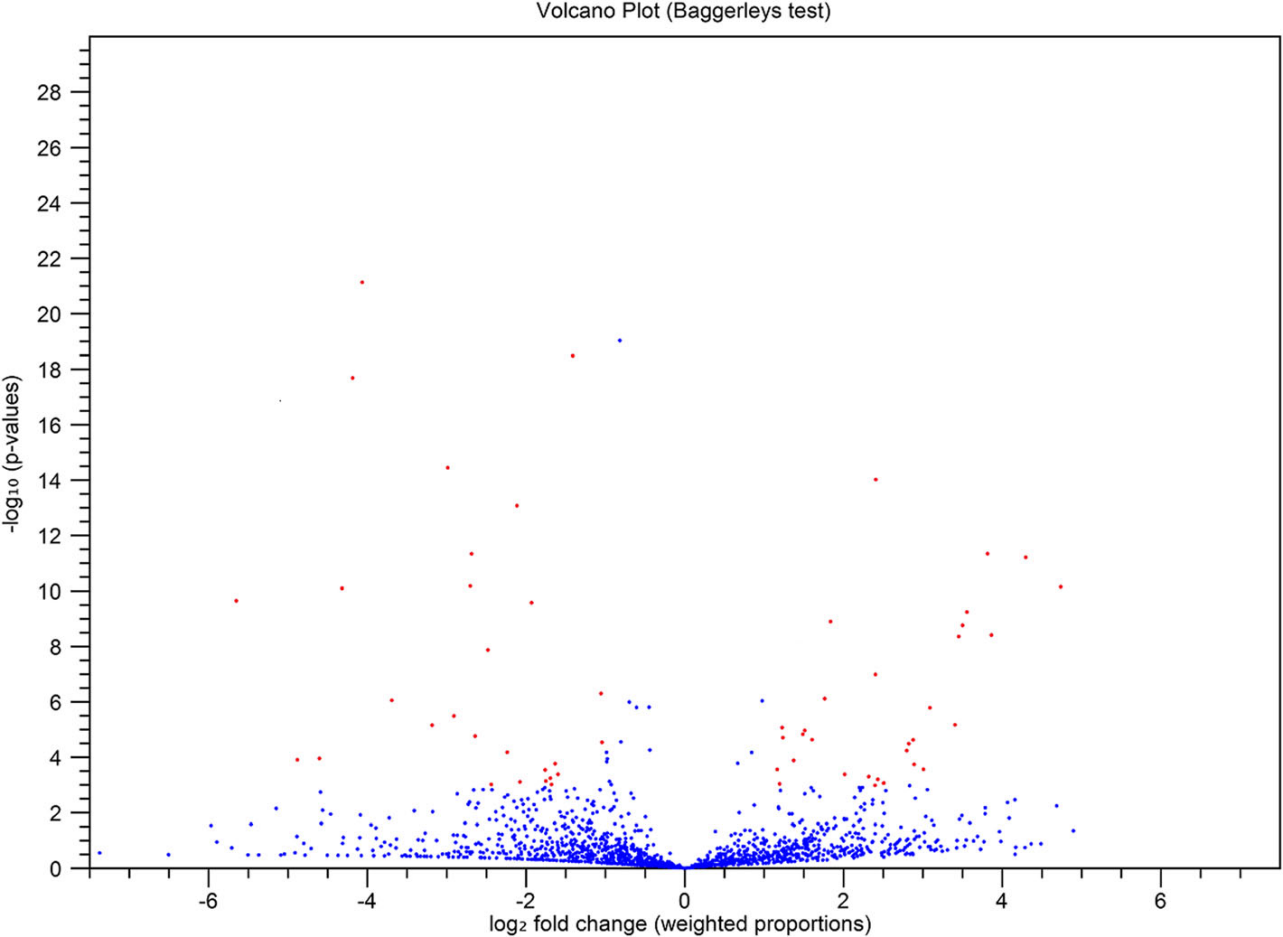
Volcano plot showing overall miRNA expression. The plot was constructed based on the log2 fold change on the x-axis and –log 10 P-values on the y-axis. The blue and red dots in the plot represent miRNAs. The blue dots at positive values on the x-axis show miRNAs that were not significantly upregulated, whereas red dots at positive values on the x-axis showed miRNAs that were significantly upregulated. The blue dots at negative values on the x-axis showed miRNAs that were not significantly downregulated, whereas the red dots at negative values on the x-axis showed miRNAs that were significantly downregulated

**Fig. 5:**
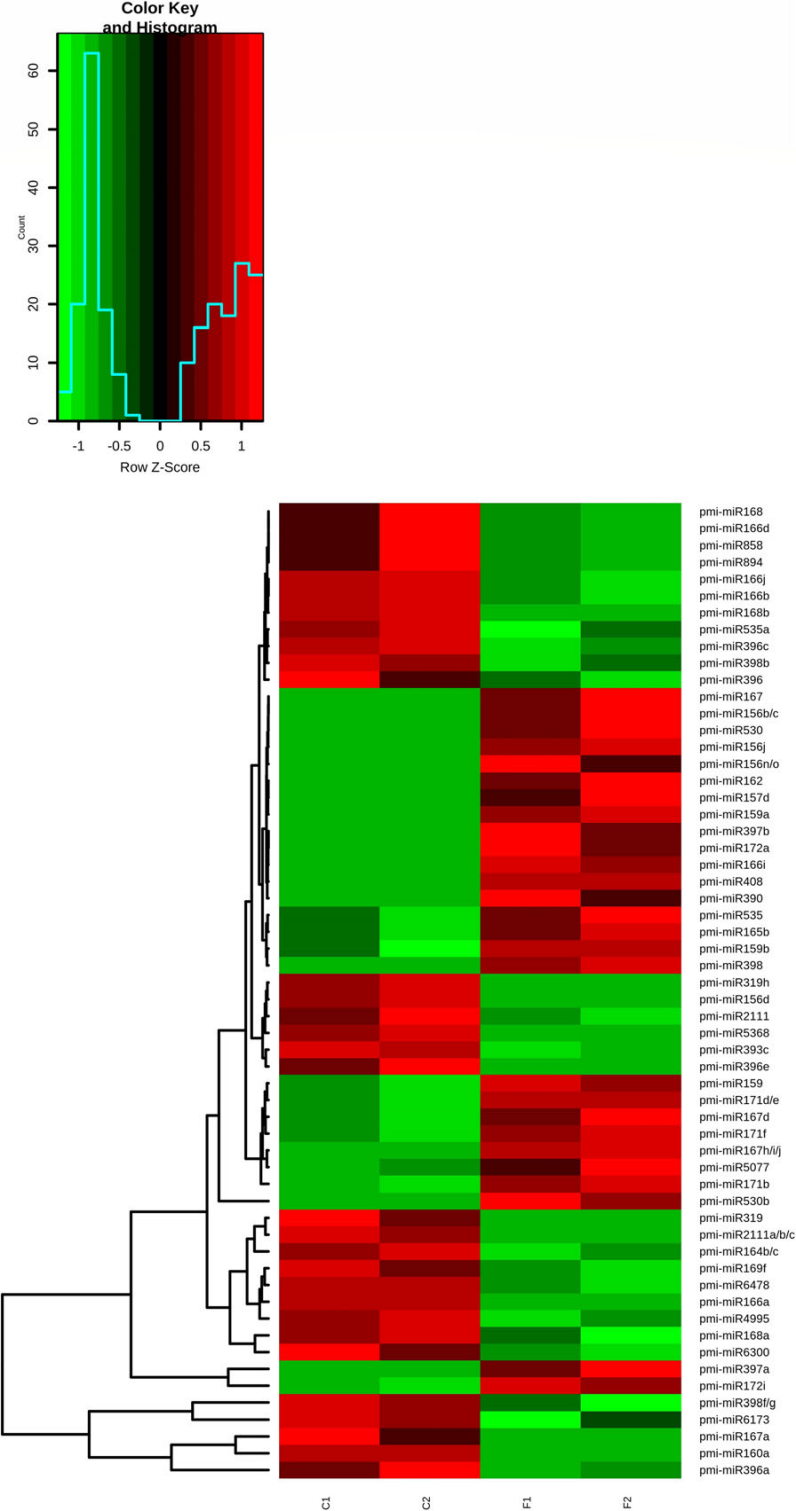
Heatmap representing miRNAs significantly altered by Baggerley’s test (*P* < 0.05) in the C and F libraries Green colour indicates low expression of miRNA, while red colour indicates high expression of miRNA

The overall expression of miRNAs in both libraries is shown in the volcano plot (Fig. 4). The plot showed significant changes in the regulation of 58 miRNAs. Among these significantly regulated miRNAs, 31 were upregulated and 27 were downregulated. Additionally, a heatmap was generated to compare the expression of 58 significantly regulated miRNAs in the C and F libraries (Fig. 5). Further details about the significantly regulated miRNAs are documented in Table 2.

**Table 2:** Statistically significant miRNA under inoculation of *F. oxysporum* (*P* < 0.05)

miRNA	Sequences (5′ to 3′)	*P*-values	FDR values
pmi-miR156b/c	TTGACAGAAGATAGAGAGCACGA	2.78E-4	0.02
pmi-miR156d	ACTCTCTGTGCTTCTGTCATCA	2.04E-18	1.88E-15
pmi-miR156j	TTGACAGAAGAGAGTGAGAA	1.12E-4	7.94E-3
pmi-miR156n/o	TGGCAGAAGAGAGTGAGCACAA	9.84E-4	4.67E-2
pmi-miR157d	CTGACAGAAGATAGAGAGCACGA	4.12E-4	2.28E-2
pmi-miR159	TTTGGATTGGAGGGAGCTCTA	4.56E-12	2.22E-9
pmi-miR159a	CTTGGATTGAAGGGAGCTA	3.50E-6	4.11E-4
pmi-miR159b	TTTGGATTGAAGGGAGCTCTCCA	5.78E-5	4.60E-3
pmi-miR160a	GCGTATGAGGAGCCAAGCATA	2.97E-120	7.50E-117
pmi-miR162	ACGATAAACCTCTGCATCCAGA	1.16E-5	1.15E-3
pmi-miR164b/c	TGGAGAAGCAGGGCACGTGCA	3.64E-7	6.23E-5
pmi-miR165b	GGAATGTTGTTTGGTTCGATGA	2.36E-5	2.11E-3
pmi-miR166a	CTCGGACCAGGCTTCATTCCCA	1.63E-31	2.35E-28
pmi-miR166b	TCGGACCAGGCTTCACTCCCAA	1.86E-7	3.36E-5
pmi-miR166d	TAGGACCAGGCTTCATTCCCTA	1.63E-4	0.01
pmi-miR166i	GGAATGTCGTCTGGTTCA	1.87E-5	1.73E-3
pmi-miR166j	TCGGACCAGGCTTCATTCCCATA	1.86E-7	3.36E-5
pmi-miR167	TGAAGCTGCCAGCATGATCTTTA	2.78E-4	0.01
pmi-miR167a	AGATCATCTGGCAGCTTCACCA	1.23E-4	8.60E-3
pmi-miR167d	TGAAGCTGCCAGCATGATCTATTA	3.84E-9	9.47E-7
pmi-miR167h/i/j	TGAAGCTGCCAGCATGATCTTA	8.54E-6	9.08E-4
pmi-miR168	TCGCTCGGTGCAGGTCGGGAA	1.63E-4	1.06E-2
pmi-miR168a	CCCGCCTTGCATCAACTGAATCA	4.69E-8	1.01E-5
pmi-miR168b	CCCGCTTTGCATCAACTGAATA	1.25E-9	3.52E-7
pmi-miR169f	TAGCCAGGGATGACTTGCCGGA	5.93E-7	9.37E-5
pmi-miR171b	TTGAGCCGTGCCAATATCACA	6.16E-12	2.71E-9
pmi-miR171d/e	TTGAGCCGTGCCAATATCACTA	2.87E-11	1.21E-8
pmi-miR171f	TTGAGCCGTGCCAATATCACAA	1.71E-9	4.55E-7
pmi-miR172a	AGAATCTTGATGATGCTGCACTA	7.68E-6	8.25E-4
pmi-miR172i	AGAATCTTGATGATGCTGCATTA	9.55E-15	6.89E-12
pmi-miR2111	TAATCTGCATCCTGAGGCCCA	1.77E-6	2.33E-4
pmi-miR2111a/b/c	TAATCTGCATCCTGAGGCTAA	8.75E-13	5.53E-10
pmi-miR319	CTTGGACTGAAGGGAGCTCCCTA	3.70E-7	6.23E-5
pmi-miR319h	CTTGGACTGAAGGGAGCTCCA	3.56E-15	2.76E-12
pmi-miR390	AAGCTCAGGAGGGATAGCGCCTA	3.69E-4	0.02
pmi-miR393c	TCCAAAGGGATCGCATTGATCCA	4.61E-12	2.22E-9
pmi-miR396	TTCCACAGCTTTCTTGAACTCCA	5.40E-4	2.90E-2
pmi-miR396a	GTTCAATAAAGCTGTGGGAAA	2.76E-4	0.02
pmi-miR396c	GTTCAAGAAAGCTGTGGGATGA	1.87E-6	2.42E-4
pmi-miR396e	TTCCTCAGCTTTCTTGAACTGA	4.62E-7	7.66E-5
pmi-miR397a	TCATTGAGTGCAGCGTTGATAA	2.32E-5	2.10E-1
pmi-miR397b	TCATTGAGTGCAGCGTTGTTGA	7.68E-6	8.26E-4
pmi-miR398	TGTGTTCTCGGGTCGCCCCTGTA	1.02E-9	3.22E-7
pmi-miR398b	TGAGTTCTCAGGTCGCCCCTA	1.07E-4	7.77E-3
pmi-miR398f/g	TGTGTCCTCAGGTCGCCCCCA	7.05E-7	1.07E-4
pmi-miR408	TGCACTGCCTCTTCCCTGGTTA	4.58E-5	3.76E-3
pmi-miR530	TATCTGCATTTGCACCTGCACCA	1.77E-4	0.01
pmi-miR530b	TGCATTTGCACCTACACCTTAA	7.14E-11	2.78E-8
pmi-miR535	TGACAATGAGAGAGAGCATA	2.73E-4	0.02
pmi-miR535a	TTTGACAAAGAGAGAGAGCACGA	9.67E-4	0.04
pmi-miR858	TTCGTTGTCTGTTCAACCTTA	1.63E-4	0.01
pmi-miR894	TTTCACGTCGGGTTCATCAA	1.63E-4	0.01
pmi-miR4995	TAGGCAGTGGCTTGGTTAAGGA	4.13E-10	1.39E-7
pmi-miR5077	TCGCGTCGGGTTCACCAA	9.20E-4	0.04
pmi-miR5368	GACAGTCTCAGGTAGACA	2.61E-10	9.11E-8
pmi-miR6173	AGCCGTAAACGATGGATA	1.714E-4	0.01
pmi-miR6300	GTCGTTGTAGTATAGTGGA	2.28E-7	4.04E-5
pmi-miR6478	TCGACCTTAGCTCAGTTGGTA	6.66E-11	2.69E-8

### Common and specific miRNAs

**Fig. 6:**
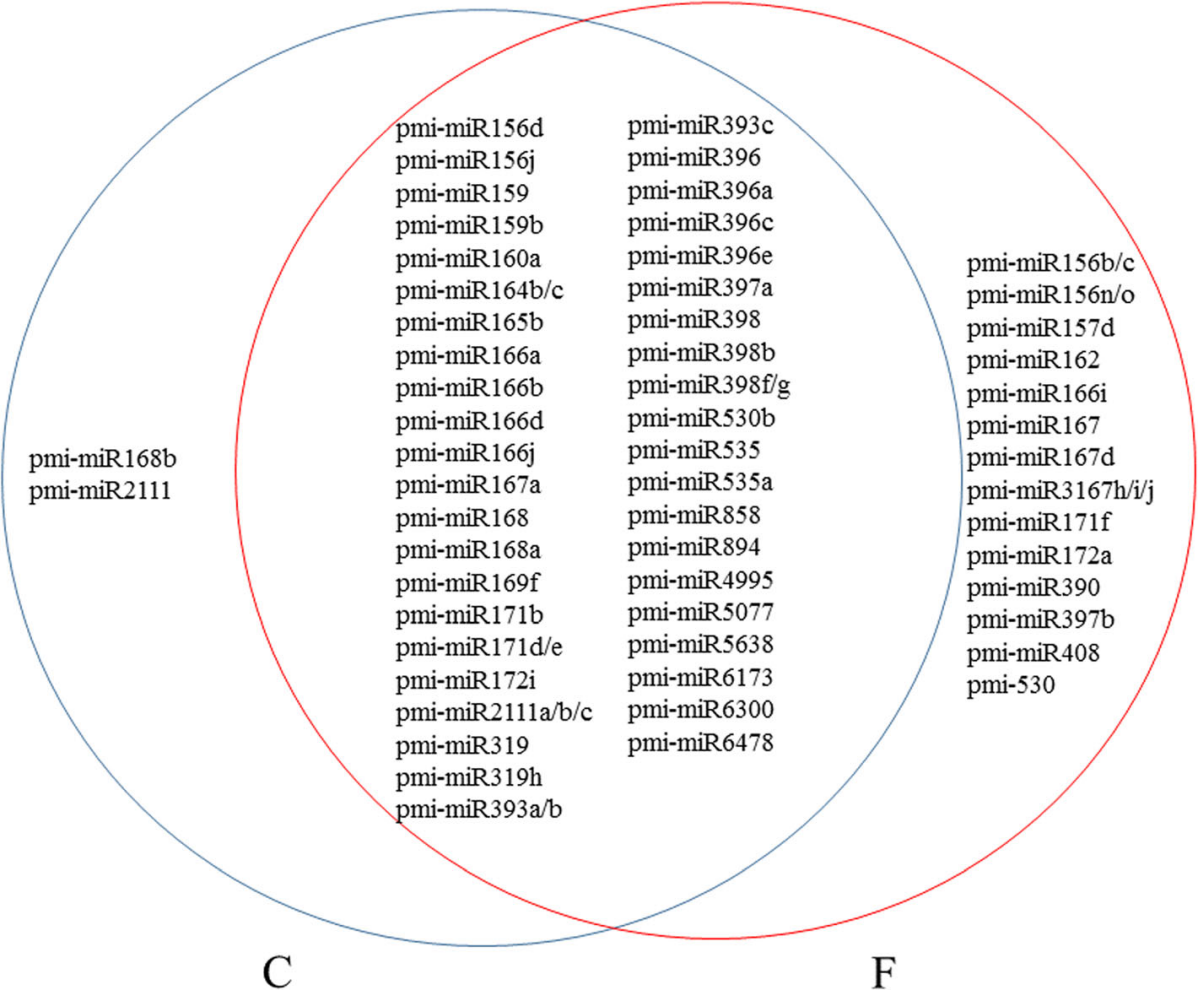
Common and specific miRNA sequences in the C and F libraries

A Venn diagram was generated to show the common and specific miRNAs in the C and F libraries (Fig. 6). Of the 58 miRNAs responsive to *F. oxysporum* treatment, 42 miRNAs were common to both libraries. Two miRNAs, pmi-miR168b and pmi-miR2111, were found to be specific to the C library, whereas 14 miRNAs were specific to the F library.

### Discovery of novel miRNAs

**Fig. 7:**
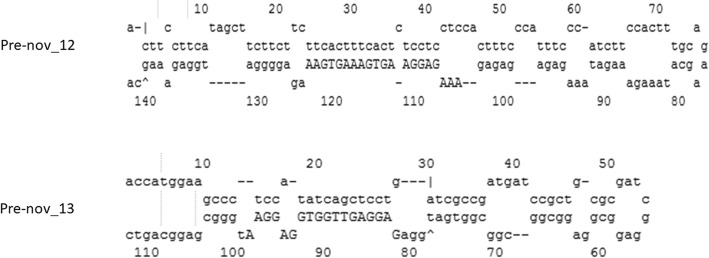
Stem-loop or pre-miRNA structures for putative novel miRNAs

**Fig. 8:**
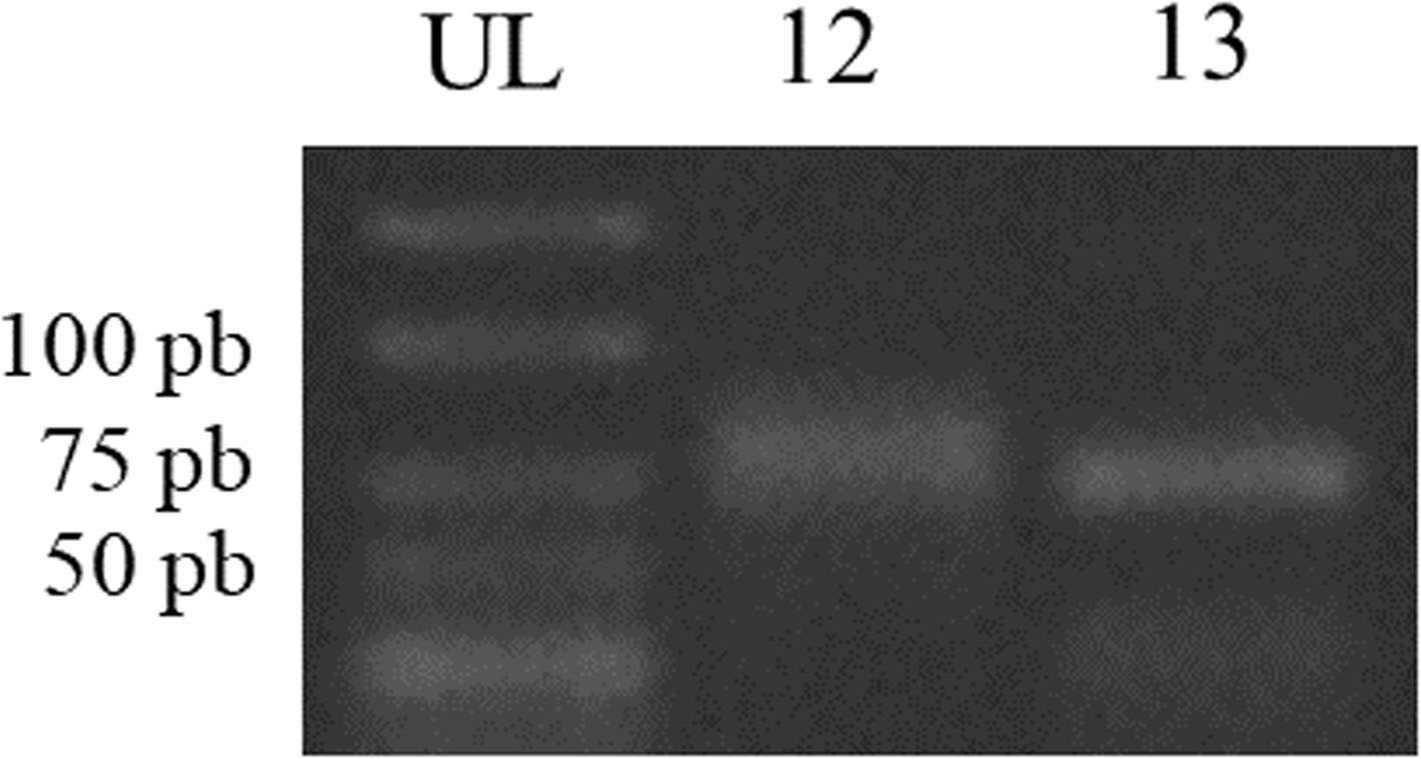
Experimental validation of miRNA stem-loop structure through RT-PCR

To discover novel miRNA sequences in *P. minor*, all unannotated small RNAs were searched against the *P. minor* transcriptome data under accession number SRX669305 [26]. After searching for hairpin structures (Fig. 7) and performing MFEI calculations (Table 3), two unique sequences were identified as putative novel miRNAs in *P. minor*. These putative novel miRNAs were named pmi-nov_miR12 and pmi-nov_miR13. The secondary structures of both miRNAs were validated through reverse transcriptase PCR (Fig. 8). The sizes of the pmi-nov_miR12 precursor (pre-nov_12) and pmi-nov_miR13 precursor (pre-nov_13) are ~ 80 bp and ~ 75 bp, respectively. The sizes of the validated precursors were slightly smaller than the predicted sizes. These smaller sizes might be due to the primers, which were designed to avoid the bulge structure in the hairpin structures. The bulge region in the hairpin structure could have reduced the efficiency of primer binding to the DNA template [27].

**Table 3:** List of novel miRNAs discoveries and their information

ID miRNA^a^	miRNA sequence (5′ to 3′)	Pre-miRNA ID^b^	Length of precursors (nt)	ID transcript^c^	% A and U	MFE (kcal/mol)^d^	AMFE^e^	MFEI^f^
Pmi-nov_12	AAAGAGGAAGTGAAAGTGAA	Pre-nov_12	143	comp65772_c1_seq1	58.04	−46.40	32.45	1.30
Pmi-nov_13	GAGGAGTTGGTGGAGGAA	Pre-nov_13	113	comp63496_c0_seq5	35.40	−49.10	43.45	1.49

^a^miRNA identification

^b^Pre-miRNA identification;

^c^Transcript identification

^d^Minimum folding energy

^e^Adjusted minimum folding energy

^f^Minimum folding energy index

### Prediction and functional annotation of target genes

**Fig. 9:**
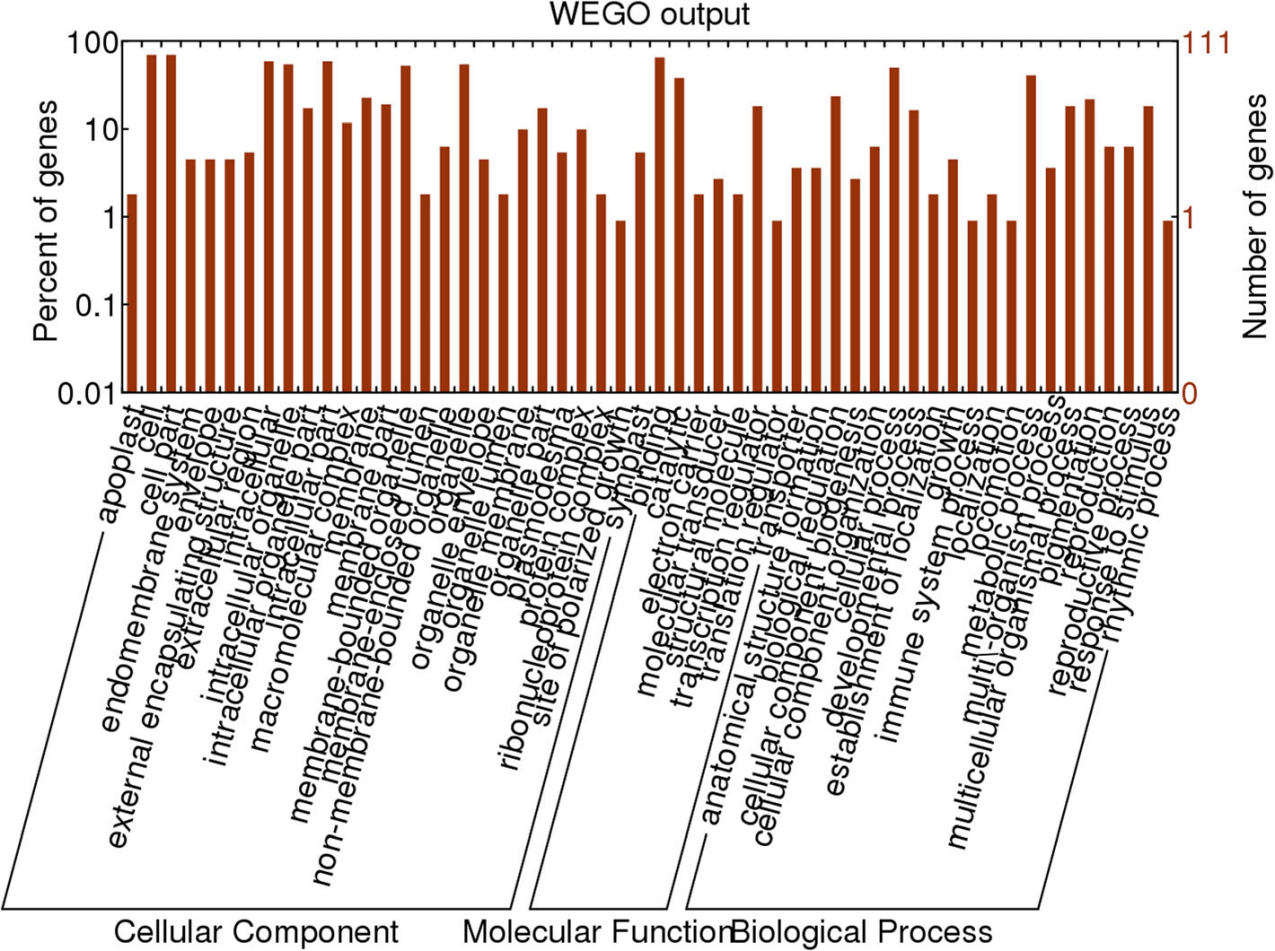
Functional classification of target transcripts by WEGO software

In plants, miRNAs are involved in many biological processes by negatively regulating their target genes/transcripts [11, 28]. In this study, we searched for putative target genes against the *P. minor* transcriptomic library software with mismatch score of 4.0 [29]. This prediction resulted in a total of 111 potential target genes in *P. minor*. Among them, there were 108 target genes for 58 conserved miRNAs and 3 for two novel miRNAs (Table 4). The majority of the miRNAs have more than one target gene. The target genes were classified according to the WEGO database into cellular component, biological process and molecular function categories (Fig. 9).

**Table 4:** Target prediction for responsive miRNA in *P. minor* against *F. oxysporum*

miRNA	Score	ID target	Target annotation
pmi-miR156b/c	1.0	comp48942_c0_seq1	SPL13
	3.0	comp66611_c0_seq1	Ferric reduction oxidase
	3.5	comp40605_c1_seq1	F-box kelch repeat
pmi-miR156d	3.5	comp52863_c0_seq1	F-box protein SKIP27
	3.5	comp64448_c1_seq76	Nuclear transcription factor Y subunit A
pmi-miR156j	2.0	comp51572_c1_seq2	SPL4
	2.0	comp54862_c0_seq3	Malate dehydrogenase
	2.5	comp40605_c1_seq1	F-box kelch repeat
pmi-miR156n/o	1.5	comp52471_c0_seq1	SPL16
	3.0	comp63113_c0_seq2	bHLH55
	4.0	comp52567_c0_seq3	Probable signal peptidase complex subunit 1
pmi-miR157d	3.8	comp65851_c2_seq1	Cytochrome P450 81F1
	4.0	comp62138_c0_seq4	Probable carboxylesterase18
pmi-miR159	1.0	comp57600_c3_seq1	Transcription factor GAMYB
	2.0	comp67380_c0_seq2	Putative disease resistance protein RGA3
pmi-miR159a	2.5	comp58874_c0_seq4	WUSHEL
	2.5	comp65589_c1_seq2	Uncharacteized protein At2g41620
pmi-miR159b	1.5	comp57600_c3_seq1	Transcription factor GAMYB
	3.8	comp59983_c0_seq2	Mitogen-activated protein kinase kinase kinase1
	4.0	comp61116_c0_seq1	Transcription factor bHLH140
pmi-miR160a	2.5	comp65542_c1_seq1	Probable galacturonosyltransferase-like 5
	3.5	comp63097_c0_seq14	Auxin-responsive protein IAA9
pmi-miR162	4.0	comp61878_c1_seq1	ABC transporter C family member3
pmi-miR164b/c	3.0	comp58722_c2_seq1	Putative disease resistance protein RGA4
	3.2	comp64889_c2_seq23	E3 ubiquitin-protein ligase RNF8-A
pmi-miR165b	3.5	comp12615_c0_seq1	Putative F-box protein At3g10240
	3.8	comp31134_c0_seq2	F-box protein At3g44326
	3.8	comp62577_c0_seq2	Probable galacturonosyltransferase12
pmi-miR166a	1.5	comp62172_c1_seq10	Homeobox-leucine zipper protein HOX32
	3.5	comp67610_c2_seq1	Probable WRKY transcription factor19
pmi-miR166b	4.0	comp66580_c3_seq9	Serine/threonine-protein kinase TIO
pmi-miR166d	2.8	comp62172_c1_seq1	Homeobox-leucine zipper protein HOX32
	4.0	comp23266_c0_seq1	Probable disease resistance protein At5g63020
pmi-miR166i	2.0	comp67539_c0_seq10	Probable LRR receptor-like protein kinase At1g51890
	2.5	comp58141_c0_seq2	NADH dehydrogenase (ubiquinone) complexI
	3.5	comp67658_c0_seq4	Programmed cell death protein4
pmi-miR166j	1.8	comp62172_c1_seq12	Homeobox-leucine zipper protein ATHB-8
	4.0	comp60418_c0_seq2	Protein caperon dnaJ15
pmi-miR167	2.5	comp65895_c0_seq14	Probable glycosyltransferase At5g03795
	3.5	comp63430_c0_seq5	Tubulin α-5 chain
pmi-miR167a	2.0	comp67548_c0_seq10	26S protease regulatory subunit 6B homolog
	4.0	comp22416_c0_seq1	Putative ribonuclease H Protein At1g65750
pmi-miR167d	4.0	comp58506_c2_seq5	Pre-mRNA-splicing factor SF2
	4.0	comp67417_c0_seq18	Binding protein DNA BIN4
pmi-miR167h/i/j	2.5	comp65003_c0_seq2	AR6 HPI
	3.2	comp52198_c0_seq1	E3 ubiquitin protein ligase DRIP1
	3.8	comp60694_c0_seq1	Probable mediator of RNA polymerase II transcription subunit 26b
pmi-miR168	3.5	comp2671_c0_seq1	Uncharacterized mitochondrial protein AtMg00820
	3.5	comp65213_c0_seq4	Putative F-box protein At3g47150
pmi-miR168a	3.5	comp67057_c0_seq6	Guard cell S-type anion channel SLAC1
pmi-miR168b	3.8	comp33471_c0_seq1	U-box domain-containing protein 36
pmi-miR169f	3.0	comp65145_c0_seq1	Serine/threonine-protein kinase ppk 15
	3.2	comp60891_c3_seq1	Nuclear transcription factor Y subunit A
pmi-miR171b	1.5	comp59653_c0_seq3	SCL6
	2.5	comp68032_c2_seq2	Putative disease resistance protein RGA4
pmi-miR171d/e	1.8	comp59653_c0_seq3	SCL6
	2.2	comp48883_c1_seq1	Uncharacterized mitochondrial protein AtMg01060
	3.8	comp58120_c1_seq1	Transmembrane protein 214-A
pmi-miR171f	2.0	comp59653_c0_seq3	SCL6
	2.5	comp68032_c2_seq3	Putative disease resistance protein RGA4
pmi-miR172a	1.5	comp52285_c0_seq1	AP2
pmi-miR172i	3.5	comp57377_c0_seq5	Transcription factor NAC29
	3.8	comp63311_c0_seq3	Transcription factor CAULIFLOWER
	4.0	comp33332_c0_seq1	E3 ubiquitin-protein ligase makorin
pmi-miR2111	3.0	comp29780_c0_seq1	Probable WRKY transcription factor 7
	3.5	comp41575_c0_seq2	Putative ribonuclease H protein At1g65750
pmi-miR2111a/b/c	2.5	comp41652_c0_seq2	Transcription factor WRKY55
	3.2	comp59219_c1_seq1	Actin-related protein 6
	3.5	comp63701_c0_seq2	Transcription factor bHLH51
pmi-miR319	3.8	comp61400_c1_seq1	Probable sulfate transporter 3.5
pmi-miR319h	4.0	comp41320_c0_seq1	Small heat shock protein
pmi-miR390	3.8	comp68004_c1_seq12	Putative disease resistance protein RGA4
	3.8	comp68227_c1_seq27	AGO5
pmi-miR393c	3.2	comp60765_c4_seq1	Proteasome subunit α type-1-A
	3.8	comp64012_c0_seq1	Pectinesterase31
pmi-miR396	2.5	comp12732_c0_seq1	Putative ribonuclease H protein At1g65750
pmi-miR396a	2.5	comp60490_c0_seq1	Peroxidase57
	4.0	comp47449_c0_seq1	Probable DXS
pmi-miR396c	2.5	comp47994_c0_seq1	Hypersensitive-induced response protein 1
pmi-miR396e	3.2	comp58032_c1_seq1	Cytochrome b
	3.5	comp60164_c0_seq2	Putative F-box protein At3g23950
pmi-miR397a	0.8	comp67947_c0_seq1	Laccase-4
pmi-miR397b	1.5	comp67947_c0_seq1	Laccase-4
pmi-miR398	3.5	comp65120_c0_seq4	L-ascorbate oxidase
pmi-miR398b	3.8	comp63418_c1_seq1	Homeobox-leucine zipper protein ANTHOCYANINLESS2
	4.0	comp34288_c0_seq1	Cellulose synthase-like protein D3
pmi-miR398f/g	4.0	comp67631_c2_seq15	DXR
pmi-miR408	1.5	comp50583_c0_seq1	Basic blue protein
pmi-miR530	4.0	comp31767_c1_seq1	MVD
	4.0	comp56913_c1_seq1	Probable sulphate transporter
pmi-miR530b	3.0	comp66662_c8_seq34	50S ribosomal protein L2
pmi-miR535	2.5	comp53737_c0_seq1	Ferredoxin-thioredoxin reduktase catalytic chain
pmi-miR535a	3.2	comp51270_c0_seq1	Transcription factor TCP8
	3.5	comp55340_c0_seq1	Probable cellulose synthase A catalytic subunit 5
pmi-miR858	3.8	comp47431_c1_seq1	E3 ubiquitin-protein ligase ATL31
	4.0	comp55416_c0_seq1	Callos synthase 10
pmi-miR894	2.0	comp66746_c0_seq1	U-box domain-containing protein 13
	2.5	comp67024_c1_seq10	Heat shock 70 kDa protein 16
	4.0	comp18439_c0_seq1	Polygalacturonase At1g48100
pmi-miR4995	3.0	comp60227_c1_seq1	Probable WRKY transcription factor 39
pmi-miR5077	3.2	comp30119_c0_seq1	Ethylene-responsive transcription factor ER0 HPI25
	3.5	comp63610_c1_seq2	Cellulose synthase A catalytic subunit 7
pmi-miR5368	3.8	comp12493_c0_seq1	Sucrose synthase 5
pmi-miR6173	3.0	comp46206_c0_seq1	Sesquiterpene synthase
	4.0	comp62238_c1_seq1	Farnesyl diphosphate synthase 1
pmi-miR6300	3.2	comp55945_c0_seq1	HMGR
	3.5	comp59913_c0_seq1	Proteasome subunit beta type-2-A
pmi-miR6478	3.8	comp62172_c1_seq14	Homeobox-leucine zipper protein HOX33
pmi-nov_12	2.5	comp58932_c3_seq6	Alcohol dehydrogenase
pmi-nov_13	2.5	comp65932_c1_seq10	Mitogen-activated protein kinase 16
	3.8	comp56286_c0_seq1	Mevalonate kinase

### Analysis of target transcripts involved in terpenoid pathway

The terpenoid pathway consists of two distinct pathways, MVA and MEP. From the target prediction (Table 4), a total of seven miRNA targets were discovered that seemed to be directly involved in these terpenoid biosynthesis pathways. In the MVA pathway, the target involved were diphosphomevalonate decarboxylase (MVD), targeted by pmi-miR530; sesquiterpene synthase and farnesyl diphosphate synthase (FDS), targeted by pmi-miR6173; 3-hydroxy-3-methylglutaryl-coenzyme A reductase (HMGR), targeted by pmi-miR6300; and mevalonate kinase (MVK), targeted by pmi-nov_13. In the MEP or non-mevalonate pathway, the identified targets were 1-deoxy-d-xylulose-5-phosphate synthase (DXS) and 1-deoxy-d-xylulose-5-phosphate reductoisomerase (DXR), which were targeted by pmi-miR396a and pmi-miR398f/g, respectively. In addition, two more targets, peroxidase and alcohol dehydrogenase (ADH), were targeted by pmi-miR396a and pmi-nov_12, respectively. Both of these targets were included in this analysis, because peroxidase is involved in the early signalling of secondary metabolite biosynthesis, and ADH is an enzyme involved in green leaf volatile (GLV) biosynthesis. GLV compounds have been reported to have similar physiological and functional properties to those of terpenoids [30, 31].

### Expression profiles of miRNAs and their targets by RT-qPCR

**Fig. 10:**
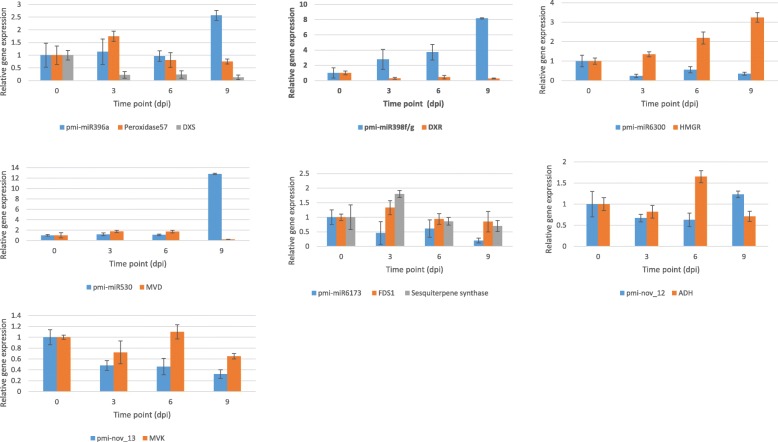
Relative expression of miRNAs with respect to their target transcripts

Real-time quantitative polymerase chain reaction (RT-qPCR) was performed to experimentally validate the expression of five conserved miRNAs (pmi-miR396a, pmi-miR398f/g, pmi-miR530, pmi-miR6173, and pmi-miR6300) and two novel miRNAs (pmi-nov_12 and pmi-nov_13) and their target transcripts. The expression profiles are shown in Fig. 10. Pmi-miR396a showed upregulation to 2.5-fold at 9 dpi. Its target, peroxidase57, was upregulated at 3 dpi and then downregulated at 6 dpi and 9 dpi. Peroxidase is an important enzyme that acts as a scavenger for reactive oxygen species (ROS) [32]. ROS are produced by plants as an early response to stress. Simultaneously, plants require a mechanism to prevent ROS from damaging the cell. Hence, high production of peroxidase during early inoculation (3 dpi) may help neutralize the excessive ROS inside the plant cell. In addition, peroxidase may also be involved in terpenoid biosynthesis. In cucumber inoculated with red spider mites (*Tetranychus urticae* Koch), increased production of (E, E)-α-farnesene resulted [33]. In *A. thaliana*, miR396 is encoded by two different gene loci, which lead to the biogenesis of two miR396 members, miR396a and miR396b, targeting the GRF transcription factor, which regulates the number of cells in the leaf [34, 35]. Overexpression of miR396a and miR396b resulted in reduced leaf size [34]. In addition, recent studies have revealed that the application of a target mimic approach in miR396a contributes to plant defence against fungal pathogens. The study suggested that the low activity of miR396a induces the plant defence mechanism through the accumulation of hydrogen peroxidase (H_2_O_2_) and callus formation [36]. In rice, a similar approach was used, leading to decreased accumulation of miR396a and increased GRF transcription factor activity. That kind of interaction activates auxin biosynthesis and ARF, thus resulting in greater yield [37]. In addition to peroxidase, another target of pmi-miR396a in *P. minor* was DXS. Negative correlations between these two were observed at 3 dpi. DXS is involved in the MEP pathway, which produces monoterpene and diterpene as the main products [9, 10]. The descending pattern of DXS expression corresponds with the increase in pmi-miR396a, especially at 3 dpi and 9 dpi. For the target transcript peroxidase57, the target was not strongly regulated with high expression of pmi-miR396a at 3 dpi. This kind of regulation was also demonstrated for miR398 in *A. thaliana* [38]*.* In *A. thaliana*, miR398 targeted two types of copper superoxide dismutase (CSD), CSD1 and CSD2. The upregulation of miR398 consistently leads to the downregulation of CSD1, whereas CSD2 did not show any correlation [38].

In *P. minor*, the expression profile of pmi-miR398f/g showed an increasing pattern from 3 dpi to 9 dpi, and the maximum was observed at 9 dpi. The upregulation of pmi-miR398f/g led to the downregulation of the DXR target transcript. Similar to DXS, DXR is involved in the MEP pathway of terpenoid biosynthesis [10]. The miR398 family is thought to be involved in various plant stress responses by participating in the oxidative burst process [39, 40]. In *A. thaliana*, the expression of miR398 was downregulated under salinity, oxidative stress, *Pseudomonas syringae* infection and phosphate deficiency. This downregulation leads to the accumulation of a target transcript, CSD, which acts as a ROS scavenger [39]. In *P. minor*, pmi-miR398f/g was upregulated upon treatment with *F. oxysporum*. The expression pattern seems to contrast with that of miR398 in *A. thaliana*. However, in *Medicago truncatula,* miR398a/b, which targets copper-containing proteins involved in copper homeostasis, was upregulated under drought conditions [41].

Pmi-miR6300 showed a decreasing pattern from 3 dpi until 9 dpi, resulting in the accumulation of the target transcript, HMGR. The highest accumulation of HMGR was observed at 9 dpi, when RT-qPCR showed an upregulation of more than three-fold. HMGR is a vital enzyme involved in sesquiterpene biosynthesis in the MVA pathway [10]. A very limited amount is known about the role of the miR6300 family in plants. In barley, miR6300 was downregulated under drought treatment [42]. In addition, miR6300 has also been discovered in chickpea [43]. However, the target transcripts of miR6300 in both the mentioned studies are still unknown.

Pmi-miR530 targeted MVD, which is involved in the MVA pathway. No significant change was observed in pmi-miR539 expression until 6 dpi. On the other hand, the target transcript MVD showed a gradual increase from 3 dpi until 6 dpi. At 9 dpi, pmi-miR530 was drastically upregulated (exceeding 12-fold), while MVD was repressed. The spike in pmi-miR530 expression was supported by a previous study in chickpea. In chickpea inoculated with *F. oxysporum*, there was a 17-fold upregulation of miR530 compared to that in control plants. In addition, miR530 in chickpea targeted zinc knuckle protein, which is involved in regulating plant development [44].

Two target transcripts, FDP and sesquiterpene synthase, which are involved in the MVA pathway, were targeted by pmi-miR6173. RT-qPCR analysis showed a decreasing pattern in pmi-miR6173 expression. The expression patterns for both targets were similar. The expression of target transcripts increased at 3 dpi. However, the expression declined at 6 dpi, and no significant change was observed from 6 dpi to 9 dpi. A decreasing expression pattern of miR6173 was also observed in the herbs *Sedum alfredii* and *Medicago sativa*. In *S. alfredii*, miR6173 was downregulated under cadmium treatment. Target prediction showed that miR6173 in *S. alfredii* targeted a number of targets, such as calcium-binding EF-hand family protein, ATP synthase subunit α, and aspartic protease. However, further work is needed to discover the role of miR6173 in *S. alfredii* [45]*.* In *M. sativa*, miR6173 was among the miRNAs downregulated under drought treatment. miR6173 in *M. sativa* targeted splicing factor 3A subunit 2, which plays an important role in mRNA splicing [46].

The single gene involved in GLV biosynthesis, ADH, was targeted by the novel miRNA pmi-nov_12. Our results showed that pmi-nov_12 was downregulated from 3 dpi to 6 dpi and then upregulated at 9 dpi, where a 1.2-fold increase was observed. The expression pattern of pmi-nov_12 was downregulated and upregulated at a series of time points, showing a dynamic expression pattern. This type of expression was previously reported in *Populus tremula.* In *P. tremula* treated with salinity and abscisic acid, miR398 was downregulated in the first 48 h and then showed high expression at 72 h. In *P. minor*, although dynamic regulation occurs in pmi-nov_12, a negative correlation can still be observed between pmi-nov_12 and ADH. ADH was reported to be involved in GLV biosynthesis by catalysing the conversion between alcohol and aldehyde via the hydroperoxide lyase pathway [47]. Another novel miRNA in *P. minor*, pmi-nov_13, was downregulated in response to *F. oxysporum* treatment, and its target transcript (MVK) was upregulated, especially at 6 dpi. MVK is involved in the MVA pathway of terpenoid biosynthesis. Notably, pmi-nov_12 and pmi-nov_13 are unique miRNAs in *P. minor* that have never been reported in another plant species before. However, for pmi-miR6173, pmi-nov_12 and pmi-nov_13, the expression of the target genes was comparable to miRNA expression even with decreasing metabolite content at the end of the treatment (9 dpi). Hence, we suggest that these miRNAs may inhibit mRNA translation, which is another mode of miRNA action [48, 49].

### miRNAs as post-transcriptional regulator in *P. minor*

**Fig. 11:**
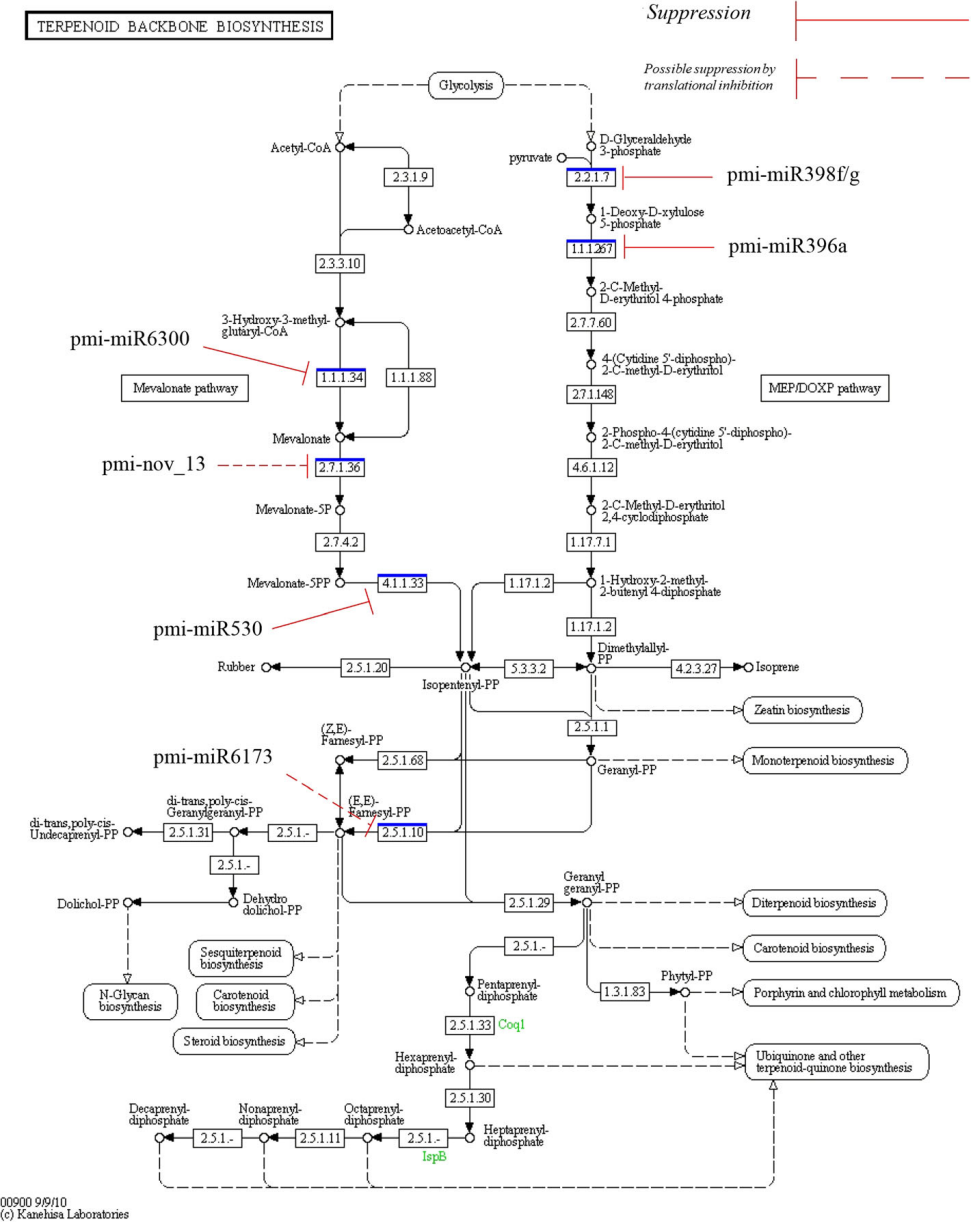
Involvement of miRNAs in the terpenoid pathway in *P. minor* EC 2.2.1.7: 1-deoxy-D-xylulose-5-phosphate synthase; EC 1.1.1.267: 1-deoxy-D-xylulose-5-phosphate reductoisomerase; EC 1.1.1.34: hydroxymethylglutaryl-CoA reductase; EC 2.7.1.36: mevalonate kinase; EC 4.1.1.33: diphosphomevalonate decarboxylase; EC 2.5.1.10: farnesyl diphosphate synthase. The terpenoid biosynthesis backbone pathway was constructed using KEGG software. Suppression symbol (continuous line) indicate the miRNAs had displayed negative relationship against their own target, while dashed suppression symbol indicate the hypothetical effect of miRNAs to inhibit the target via translational inhibition

In plants, miRNAs play roles in various biological processes, such as plant development, signal transduction, stress response, and secondary metabolite regulation [50]. In this study, miRNAs were discovered that regulate both the MVA and MEP pathways in terpenoid biosynthesis in *P. minor* (Fig. 11). Four miRNAs, pmi-miR6300, pmi-nov_12, pmi-miR530, and pmi-miR6173, were found to be involved in the MVA pathway, whereas two miRNAs, pmi-miR396a and pmi-miR398f/g, were found to be involved in the MEP pathway. Most of the miRNAs regulate upstream genes in the terpenoid biosynthesis pathway.

In the MVA pathway, among the four responsive miRNAs, three (pmi-miR6300, pmi-6173 and pmi-nov_13) were downregulated, whereas the fourth miRNA (pmi-miR530) showed a sharp increase at 9 dpi. The Pmi-miR6300-regulated gene encoded HMGR, a rate-limiting enzyme in the MVA pathway and the first rate-limiting enzyme recognized in the terpenoid biosynthesis pathway [51]. In *A. thaliana*, the HMGR gene has undergone a duplication process that led to HMG1 and HMG2. HMG1 is expressed throughout the plant, whereas the expression of HMG2 was observed in only the meristem and floral parts of *A. thaliana* plants [51]. In addition, the expression of HMGR is affected by internal factors such as plant development and phytohormone [52]. HMGR is also affected by external stimuli, such as light, wounding, elicitor treatment and pathogen invasion [52, 53]. The mutant *hmg*1 in *A. thaliana* exhibited infertility, premature senescence, and dwarfness [54]. In the MVA pathway, HMGR catalyses the rate-limiting steps that directly affect downstream products. In *A. thaliana*, the *hmg*1 mutant showed a 65% reduction in triterpene compound accumulation compared to the wild type [55]. Moreover, elicitor treatments such as fungal inoculation and wounding also increased the expression level of HMGR and subsequently led to higher accumulation of sesquiterpene compounds [53, 56]. In *P. minor*, terpenoids are produced in small quantities under normal conditions. RT-qPCR results showed that pmi-miR6300 was upregulated at 0 dpi, which led to the suppression of its target, HMGR. Indeed, after treatment, pmi-miR6300 was downregulated and led to the high accumulation of HMGR. In *X. strumarium*, HMGR was targeted by miR1134 and miR5021 [50]. However, the study of both miRNA and HMGR in *X. strumarium* is still at the prediction level and requires further experimental validation. In the MVA pathway, the HMGR enzyme catalyses the conversion of HMG-CoA to mevalonate, which is later converted into mevalonate-5-phosphate through the enzyme MVK. Until now, no clear interaction between miRNA and MVK has been reported in plants, while in mice, miR122 targets MVK, which is involved in cholesterol biosynthesis [1, 57]. In this study, we investigated the interaction and reported that pmi-nov_13 targets MVK in *P. minor*. The second miRNA, pmi-miR530, was found to regulate the MVD enzyme, which catalyses mevalonate diphosphate into IPP. Pmi-miR530 was increased drastically at 9 dpi, which led to the downregulation of its target. As for pmi-nov_13 and MVK, no miRNA in plants has previously been reported to target MVD. In mice, miR124 is involved in hypocholesterolaemia by targeting MVD [58]. In plants, information about the roles of MVK and MVD is quite limited compared to yeast [59]. However, the latest study of MVK in *Gingko biloba* discovered that overexpression of MVK led to the accumulation of the terpene trilactone [60]. In *P. minor*, the accumulation of MVK at 6 dpi may contribute to sesquiterpene biosynthesis, although the rate-limiting enzyme had its highest expression at 9 dpi.

In the MEP pathway, two miRNAs (pmi-miR396a and miR398f/g) were discovered to be involved by targeting DXS and DXR. DXS is involved in the upstream reaction in the MEP pathway by catalysing the condensation process between pyruvate and D-glyceraldehyde 3-phosphate to produce DXP [61]. The discovery of DXS was first reported in *E. coli* and then in *A. thaliana* via mutation of the *cla* gene [61, 62]. Transgenic *A. thaliana* overexpressing the DXS gene resulted in an abundance of terpenoids compared to wild plants, and DXS was categorized as a rate-limiting enzyme in the MEP pathway [61]. Further study of DXS in *A. thaliana* showed that DXS exists as the paralogues DSX1 and DXS2. Of these two paralogues, only DXS1 was found to be involved in terpenoid biosynthesis [63].

In *A. thaliana*, the majority of the DXS genes were expressed in photosynthetic organs, such as leaves, and floral parts, while homologues to DXS2 in tomato were expressed in roots and trichomes [64]. In the second step of the MEP pathway, the DXR enzyme converts DXP to MEP via rearrangement of the molecular structure followed by a reduction process by NADPH. This step is reversible. Unlike DXS, the role of DXR as a rate-limiting enzyme is still unclear, and its function may depend on plant species, organ and development stage [65]. However, similar to DXS, DXR genes are distributed in different plant parts and stimulated by light response [65]. The mutant *dxr* exhibited an albino phenotype, defects in gibberellin and abscisic acid biosynthesis and improper formation of trichomes and stomatal closure [66].

Interestingly, in *P. minor*, the majority of miRNAs involved in the MVA pathway were downregulated except for pmi-miR530, which was upregulated at 9 dpi. The decreasing pattern exhibited by pmi-miR6300, pmi-nov_13 and pmi-miR6173 resulted in the accumulation of their target transcripts, which most likely regulate sesquiterpene production. These findings were supported by the metabolite profile in Fig. 2, which shows prominent production of α-cedrene, valencene, and β-bisabolene after treatment with *F. oxysporum*. Meanwhile, the increasing pattern shown by pmi-miR396a and pmi-miR398f/g resulted in the suppression of DXS and DXR in the MEP pathway. The differences in miRNA expression involved in regulating MVA and MEP are probably due to the effect of *F. oxysporum,* which stimulates terpenoid production in the MVA rather than the MEP pathway. Downstream products in the MVA pathway (sesquiterpene), which play crucial roles in plant defence mechanisms, may influence the selection of the terpenoid pathway in the plant system. In addition, the interaction of two regulators, miRNAs and transcription factors, can also affect terpenoid biosynthesis. In *A. thaliana* and *P. cablin*, expression of miR156 reduced the production of sesquiterpene by suppression of the target, SPL [67]. In *P. minor*, pmi-miR156b/c was predicted to target the SPL gene. However, there is no evidence that this interaction would affect sesquiterpene production, because a previous report showed that the interaction between miR156 and SPL is conserved only in regulating floral development [11, 68].

### miRNA cleavage site determination by degradome analysis

**Fig. 12:**
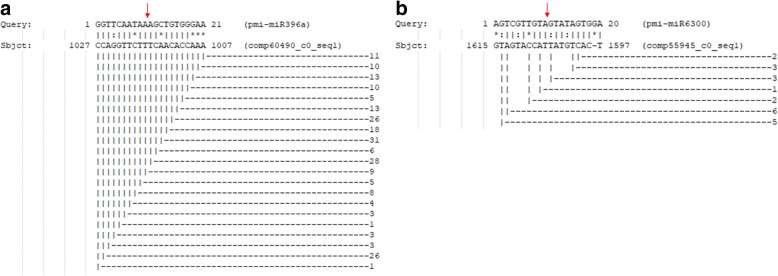
Detection of miRNA cleavage sites. The number of sequence tags is shown on the right side. The arrows indicate the positions where the miRNAs cleave the targets. **a** shows the cleavage site for pmi-miR396a, while **b** shows the cleavage site for pmi-miR6300

Degradome sequencing was carried out to determine the miRNA cleavage site. The sequences were deposited under accession number SRX3921398. The statistical data are shown in Table 5. There were 17,532,759 degradome sequencing reads. Filtering and removal of adaptors revealed 15,493,710 clean reads. Mapping to the *P. minor* transcriptome resulted in 3,079,840 sequence tags. However, to increase the hit search, another *P. minor* degradome library, with the accession number SRX3921610, was used together with this degradome library. The results showed that out of the 7 studied miRNAs involved in the terpenoid biosynthesis pathway, cleavage sites for pmi-miR396a and pmi-miR6300 and their targets, peroxidase57 and HMGR, were detected between positions 10 and 11 of the miRNA sequences (Fig. 12), and this result was also supported by previous findings [28, 29].

**Table 5:** Statistic for degradome sequencing

	Total reads	Percentages (%)
Raw reads	17,532,759	
Clean reads	15,493,710	100.00
Sequence tag	3,074,840	19.85
Discard reads	12,418,870	80.15

### Spearman correlation analysis

**Fig. 13:**
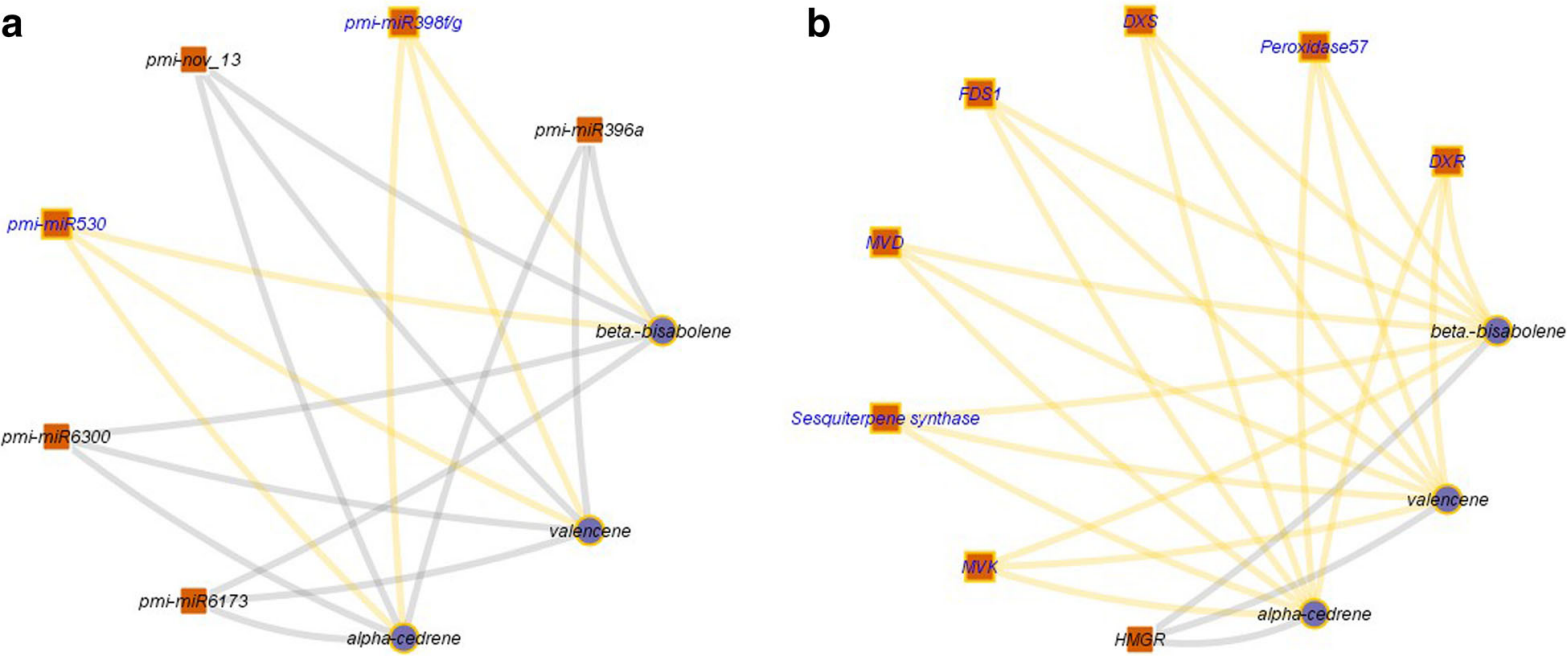
Spearman correlation analysis. Correlation analysis between miRNA-metabolites (**a**) and mRNA-metabolites (**b**). In A, miRNAs are represented by squares, whereas metabolites are represented by circles. In B, mRNAs are represented by squares, whereas metabolites are represented by circles. In both figures, yellow lines indicate positive correlations, and grey lines indicate negative correlations

Overall, correlation analysis revealed that pmi-miR530 and pmi-miR398f/g had positive correlations with metabolite content, whereas the remaining miRNAs (pmi-396a, pmi-miR6300, pmi-miR6173 and pmi-nov_13) showed negative correlations (Fig. 13a). Pmi-nov_12 was excluded from this analysis because it was not involved in the terpenoid biosynthetic pathway. For the target genes, the majority of them displayed positive correlations with metabolite content, except for HMGR (Fig. 13b). The low abundance of α-cedrene, valencene and β-bisabolene at 9 dpi may have contributed to this negative correlation because HMGR expression continued to increase during the fungus treatment. The Spearman coefficients are included in Additional file 3.

## Conclusion

In this study, a total of 58 conserved and two novel miRNAs responsive to *F. oxysporum* treatment in *P. minor* were identified. Five of the 58 conserved miRNAs and a novel miRNA were found to be directly involved with the MVA and MEP pathways. However, some of the miRNAs, such as pmi_miR6173, pmi-nov_12 and pmi-nov_13, might need further confirmation at the protein level against their own targets. This work provides the framework for further exploration of miRNA-mediated regulatory mechanisms in terpenoid biosynthesis in *P. minor*. Moreover, our study also found some miRNAs targeting mRNAs encoding transcription factors, which suggested the role of miRNAs in regulating various biological processes, including plant growth and development. The above seven studied miRNAs could be utilized to regulate secondary metabolite biosynthesis by manipulating the MVA and MEP pathways through the RNAi mechanism.

## Methods

### Plant and fungus cultures

*P. minor* plants were cultured for approximately 6 weeks on Murashige and Skoog media in a controlled chamber room at Kompleks Rumah Tumbuhan, Universiti Kebangsaan Malaysia (3° 16′ 14.63″ N, 101° 41′ 11.32″ E) [69]. The *F. oxysporum* fungal culture was obtained from a microbe collection room at the School of Biosciences and Biotechnology, Universiti Kebangsaan Malaysia. Small pieces of the fungal culture were cut and transferred to PDA plates. Two-week-old *F. oxysporum* culture was used for the inoculation.

### *F. oxysporum* inoculation and terpenoid profiling analysis

Prior to treatment, *P. minor* plants were transferred from solid to liquid MS media. The plant culture was maintained in a controlled chamber overnight. *F. oxysporum* spores were collected by rinsing the PDA with sterile distilled water. Approximately 0.05% of Tween 20 was added to the spore solution. The concentration of *F. oxysporum* was adjusted to 5 × 10^7^/mL before being added to the plant culture. The treated plants were kept in a controlled room with 16 h light and 8 h dark.

Metabolite profiling in *P. minor* was carried out at a series of time points, beginning with 3 days post-inoculation (dpi), 6 dpi, and 9 dpi, and using SPME-GCMS. In addition, *P. minor* plants treated with sterile distilled water were used as a control (0 dpi). Every time point had three biological replicates. The SPME method was adapted for the collection of volatiles. *P. minor* leaves were harvested (approximately 1 g) in liquid nitrogen and ground into small pieces. The ground tissues were then promptly transferred into labelled SPME vials to avoid evaporation of volatile compounds. The vials were closed with screw caps and then heated at 65 °*C* for 15 min in a water bath. Volatile compounds were collected by introducing an SPME needle via a septum cap. SPME fibre (100 μM polydimethylsiloxane, PDMS), which was located inside the SPME needle, was injected into the vials to absorb the volatiles. Alternatively, polyacrylate fiber also could be used for volatiles detection [70]. The equilibrium time for SPME fibre in the collection vial was set for 30 min at 55 °C–60 °C. GC-MS analysis was performed on an Agilent 7890A gas chromatograph (GC) directly coupled to the mass spectrophotometer (MS) of an Agilent 5975C inert MSD with a triple axis detector. The column used was a non-polar column, HP-5MS (30 m length × 0.25 mm) diameter and film thickness 0.25 μM. Helium was used as the carrier gas, with a flow rate of 1.3 mL/min. A splitless injection was set at 50 °C hold for 3 min, increased to 100 °C at a rate of 20 °C/ minutes, and held at 250 °C for 3 min. The peaks were identified by searching the NIST/EPA/NIH mass spectral library (version 11), and the results were combined in a GC-MS chromatogram.

### Statistical analysis of metabolite profiling

The metabolite profiling was analysed using MetaboAnalyst software (http://www.metaboanalyst.ca) version 3.0 [71]. The data were normalized according to the sum from each sample. One-way ANOVA was carried out followed by Fisher’s LSD test. The *P*-value was set at *P* < 0.05.

### Small RNA library construction

To generate high-quality small RNA libraries, the crucial step is to obtain high-quality total RNA. Low-quality starting material can reduce the quantity of small RNA [72]. Total RNA was extracted, and the quality (purity and concentration) was measured using a Nanodrop spectrophotometer (ND-1000) and Qubit, respectively. The integrity of the extracted RNA was determined by Bioanalyzer analysis (Agilent 2100) using an RNA 6000 chip. RNA samples with RNA integrity number (RIN) values over 7.0 were selected for small RNA library construction. All quality control steps were enclosed in Additional file 2. Mock-inoculated (C) and *Fusarium*-treated (F) small RNA libraries were constructed with two biological replicates, C1 and C2, and F1 and F2, respectively. For the F library, the time point at which *P. minor* emitted volatiles at the maximum level was selected for small RNA library construction. Mock-inoculated *P. minor* were prepared by adding sterile distilled water to the plant and used as a control. RNA from both the C and F samples was extracted using PureLink® Plant RNA Reagent (Thermo Fisher Scientific, USA) using the recommended protocol. Briefly, approximately 0.1 g of leaf sample from *P. minor* was ground and transferred to 1.5 mL. A volume of 0.5 mL of PureLink® Plant RNA Reagent was added and vortexed. The samples were incubated at room temperature for 5 min. Next, the samples were centrifuged at 12,000×g for 2 min at 4 °*C*. Approximately 450 μL of supernatant was transferred into new tubes, and 0.1 mL of 5 M NaCl and 0.3 mL of chloroform were added and mixed to the supernatant. The mixtures were centrifuged at 12,000×g for 2 min at 4 °*C*, and approximately 350 μL of supernatant was transferred to new tubes, mixed, and incubated for 10 min at room temperature. The tubes were then centrifuged at 12,000×g for 2 min at 4 °*C*, and a small pellet was observed at the bottom of each tube. The supernatant was discarded, and 75% ethanol was added to wash the pellet. Then, the tubes were centrifuged again at 12,000×g for 1 min at 4 °*C*. The ethanol was discarded, and the pellet was diluted with 20 μL of nuclease-free water. Then, DNase treatment was carried out for each RNA sample. The concentration of RNA was determined using a Nanodrop spectrophotometer (Thermo Scientific, USA). The integrity of the RNA was assessed by running a 1% agarose gel and Bioanalyzer analysis (Agilent 2100 Bioanalyzer, USA). A small RNA library was constructed using the NEBNext Small RNA Library preparation kit as reported by a previous study [22]. Then, the libraries were sent to Universiti Malaya, Malaysia for sequencing using an Illumina HiSeq 2500™ in Rapid Run mode.

### Bioinformatic analysis

Raw data were analysed using CLC Genomics Workbench version 8 [69]. The adaptors were removed, and the low-quality reads were filtered. Only clean reads with lengths between 18 nt and 30 nt were selected for further analysis. The annotation process was carried out by mapping the clean reads to the miRNA and non-coding-RNA databases miRBase version 21 and Rfam version 12 [23, 73]. A maximum of two mismatches were allowed [74]. Then, each library was 11normalized to transcripts per million (TPM); normalized expression = (actual miRNA count/total count of clean reads) × 1,000,000 [75]. Differential gene expression was calculated using Baggerley’s test [76]. Fold change was calculated using |log2 fold change| ≥ 2, and the *P*-value was set at *P* < 0.05 [77].

### Novel miRNA prediction

Novel miRNA prediction was carried out based on a previous study [78]. For secondary structure validation, forward and reverse primers were designed using the PrimerQuest Tool by Integrated DNA Technologies (https://sg.idtdna.com/), and the parameters were set to PCR 2 Primer (Table 6). The primer was synthesized at First Base (Malaysia). The PCR products were measured and visualized using 4% agarose gel.

**Table 6:** List of primer for pre-miRNA validation of putative novel miRNA

Pre-miRNA	Sequence (5′ to 3′)
Pre-nov_12	5′-CAC TTT CAC TCT CCT CCT CCA-3′ (Forward)
	5′-CCT CTT TCA CTT TCA CTT CCT CTT T-3′ (Reverse)
Pre-nov_13	5′-AAG CCC TCC ATA TCA GCT CCT GAT-3′ (Forward)
	5′-CCC ATT CCT CCA CCA ACT CCT-3′ (Reverse)

### Prediction of target transcript and gene ontology

PsRobot software was deployed to predict miRNA targets with a score of 4.0 to obtain more targets [29, 79]. The miRNA libraries generated in this study and the transcriptomic library of *P. minor* (http://www.ncbi.nlm.nih.gov/sra/SRX669305) were used in this target prediction. Then, the target genes were classified through gene ontology analysis using WEGO software (http://wego.genomics.org.cn/) [80]. The target genes involved in the terpenoid pathway were simply determined by the gene annotations in the transcriptomic library [81]. The genes were mapped with the KEGG database (http://www.genome.jp/kegg/) to identify the involvement of these genes in the terpenoid pathway [82].

### Validation and expression profile using real-time quantitative PCR (RT-qPCR)

To validate the existence and expression of those miRNAs and target genes involved in the terpenoid pathway under *F. oxysporum* inoculation, RT-qPCR was carried out using Maxima SYBR Green qPCR Master Mix (Thermo Fisher, USA) at a series of time points: 0 dpi, 3 dpi, 6 dpi, and 9 dpi. For miRNA, the miRNA sequence was used as the forward primer, and the universal primer from Qiagen was used as the reverse primer (Table 7). For the target genes, forward and reverse primers were designed using the PrimerQuest Tool by Integrated DNA Technologies (https://sg.idtdna.com/), and the parameters were set to PCR 2 Primer intercalating dye (Table 8). 5.8 s rRNA and tubulin were used as reference genes for the miRNAs and target genes, respectively. Relative gene expression was calculated using the Livak method [83].

**Table 7:** List of miRNA primers for RT-qPCR

miRNA	Sequence (5′ to 3′)
5.8 s rRNA	5′-ACG TCT GCC TGG GTG TCA CAA-3′ (Forward)
Pmi-miR396a	5′-GTT CAA GAA AGC TGT GGG A-3′ (Forward)
Pmi-miR6173	5′-GGG GGA GCC GTA AAC GAT GGA TA-3′ (Forward)
Pmi-miR398f/g	5′-TGT GTC CTC AGG TCG CCC CCA-3′ (Forward)
Pmi-miR530	5′-TAT CTG CAT TGT CAC CTG CAC CA-3′ (Forward)
Pmi-miR6300	5′-GGG GGT GGT TGT AGT ATA GTG GA-3′ (Forward)
Pmi-nov_12	5′-GAA AGA GGA AGT GAA AGT GAA-3′ (Forward)
Pmi-nov_13	5′-GAG GAG TTG GTG GAG GAA-3′ (Forward)

**Table 8:** List of target genes primer for RT-qPCR

Target genes	Sequence (5′ to 3′)
Tubulin	5′-TAC CAG CCA CCA ACC GTA GTC C-3′ (Forward)
	5′-CCA ACC TCC TCG TAG TCT TTC TCA A-3′ (Reverse)
Peroxidase57	5′-GGA ACC CAA ACC ACA ACT TTC-3′ (Forward)
	5′-CTG TCG CCA ATC TTT CAT CAA TC-3′ (Reverse)
DXS	5′-GGC GAA TTT GAA CTG GGT TG-3′ (Forward)
	5′-GAT TTA GCT TGT GCT TGG ATG G-5 (Reverse)
Sesquiterpene synthase	5′-AGA CGT AGT GAG CAA CCA AC-3′ (Forward)
	5′-CTT GGC ATA CCC TTG TGG TAA-3′ (Reverse)
FDS1	5′-GGG ACG ATA CTT CTC GCA AT-3′ (Forward)
	5′-GAG TGC ACT GGC TTG AAA GA-3′ (Reverse)
DXR	5′-GAC GTT TAA AGC CCC AGA CA-3′ (Forward)
	5′-AGG TCA GCT CAA CAA CCT TGA-3′ (Reverse)
MVD	5′-GCT TCA TTG AGA AAT GGA ACC G-3′ (Forward)
	5′-AAC CTT CCT ATT ACG TGC GAT TA-3′ (Reverse)
HMGR	5′-GCC AAC ATT GTG TCT GCT ATC-3′ (Forward)
	5′-ATG GTC ACG GAG ATG TGA AG-3′ (Reverse)
ADH	5′-TTA GGC GGA AGA ACA CTC AAG-3′ (Forward)
	5′-CCA ACT TGA TCT CCT GGT TAA GA-3′ (Reverse)
MVK	5′-AAG GTA AAC GCT CCG ATT CC-3′ (Forward)
	5′-CAA TGC CGC GAG AAT TTG ATT A-3′ (Reverse)

### Degradome sequencing

To determine the miRNA cleavage site, RNA samples from C and F were pooled together and sent to BGI (China) for degradome sequencing. After retrieving the raw data, low-quality reads were discarded, and adaptors were removed using Skewer software [84]. Once again, psRobot was deployed to map the degradome sequence against the reference gene (*P. minor* transcriptome) and to determine the cleavage site [29].

### Correlation analysis

To obtain overall visualization of the relationship between miRNA metabolites and mRNA metabolites, non-parametric Spearman’s rank correlation analysis was carried out using miRTarVis software (http://hcil.snu.ac.kr/~rati/miRTarVis/index.html) with the default settings [85].

## Additional files

Additional file 1: SPME GC-MS data from the inoculation test. These data show the effect of *F. oxysporum* treatment on terpenoid content in *P. minor* plants at 0, 3, 6, and 9 dpi. (CSV 1 kb)

Additional file 2: Measurement of RNA integrity for each sample. An RNA integrity check was carried out prior to small RNA library construction. (PPTX 148 kb)

Additional file 3: Spearman coefficients for miRNA-metabolite and mRNA-metabolite. Positive values indicate positive correlations, whereas negative values indicate negative correlations. (XLSX 8 kb)

## Data Availability

The raw data of small RNA libraries were deposited in NCBI under accession number SRX2645686, SRX2645687, SRX2645684, SRX2645685. The transcriptome data can be access in NCBI under accession number SRX669305. The degradome sequencing data also were submitted to NCBI under accession number SRX3921398 and SRX3921610.

## Abbreviations

ADH: Alcohol dehydrogenase
DXR: 1-deoxy-d-xylulose-5-phosphate reductoisomerase
DXS: 1-deoxy-d-xylulose-5-phosphate synthase
FDS: Farnesyl diphosphate synthase
GLV: Green leaf volatile
HMGR: 3-hydroxy-3-methylglutaryl-coenzyme A
MEP: Methylerythritol 4-phosphate
miRNA: MicroRNA
MVA: Mevalonate MVD Diphosphomevalonate decarboxylase
MVK: Mevalonate kinase
nt: Nucleotide
