# Correction to: Regulation of terpenoid biosynthesis by miRNA in *Persicaria minor* induced by *Fusarium oxysporum*

**DOI:** 10.1186/s12864-019-5994-5

**Published:** 2019-08-01

**Authors:** Abdul Fatah A. Samad, Reyhaneh Rahnamaie-Tajadod, Muhammad Sajad, Jaeyres Jani, Abdul Munir Abdul Murad, Normah Mohd Noor, Ismanizan Ismail

**Affiliations:** 1 School of Biosciences and Biotechnology, Faculty of Science and Technology, Universiti Kebangsaan Malaysia, UKM, 43600 Bangi, Selangor Malaysia; 2 Institute of Systems Biology, Universiti Kebangsaan Malaysia, UKM, 43600 Bangi, Selangor Malaysia; 3 Borneo Medical and Health Research Centre, Faculty of Medicine and Health Sciences, Universiti Malaysia Sabah, Kota Kinabalu, Malaysia; 4 Department of Plant Breeding and Genetics, University College of Agriculture and Environmental Sciences, The Islamia University of Bahawalpur, Punjab, Pakistan; 5 Department of Biosciences, Faculty of Science, Universiti Teknologi Malaysia, 81310 Skudai, Johor Malaysia

**Correction to: Samad et al. BMC Genomics (2019) 20:586**

**https://doi.org/10.1186/s12864-019-5954-0**

Following publication of the original article [1], the authors reported a number of errors, which are listed in this Correction article. The corrections are marked in bold.

1) In the Results section of the Abstract the sentence “Among these 58 differentially expressed genes (DEGs), 33 miRNAs were upregulated, whereas 25 miRNAs were downregulated” should read “Among these 58 differentially expressed genes (DEGs), **27** miRNAs were upregulated, whereas **31** miRNAs were downregulated”.

2) In the sub-section ‘Differential expression of miRNAs in C and F libraries’ the following sentences should read “The plot showed significant changes in the regulation of 58 miRNAs **with their isomiRNAs**. Among these significantly regulated miRNAs, **27** were upregulated and **31** were downregulated”.

3) In the sub-section ‘Analysis of target transcripts involved in terpenoid pathway’, “sesquiterpene synthase and farnesyl diphosphate synthase (FDS), targeted by pmi-miR6173” should instead read “sesquiterpene synthase and farnesyl diphosphate **synthase 1 (FDS1)**, targeted by pmi-miR6173”.

4) In the sub-section ‘Expression profiles of miRNAs and their targets by RT-qPCR’ the sentence “Two target transcripts, FDP and sesquiterpene synthase, which are involved in the MVA pathway, were targeted by pmi-miR6173” should instead read “Two target transcripts, **FDS1** and sesquiterpene synthase, which are involved in the MVA pathway, were targeted by pmi-miR6173”.

5) In the sub-section ‘Expression profiles of miRNAs and their targets by RT-qPCR’ the following citation [2] should have appeared after the following sentence: “This type of expression was previously reported in *Populus tremula*”.

6) The final sentence of the ‘Conclusions’ section should read: “The above **six terpenoid related miRNAs** could be utilized to regulate **terpenoid** biosynthesis by manipulating the MVA and MEP pathways through the RNAi mechanism.”

7) The authors have provided corrected versions of Figs. 4 and 11, which are reproduced with the same figure legends below.

**Fig. 4:**
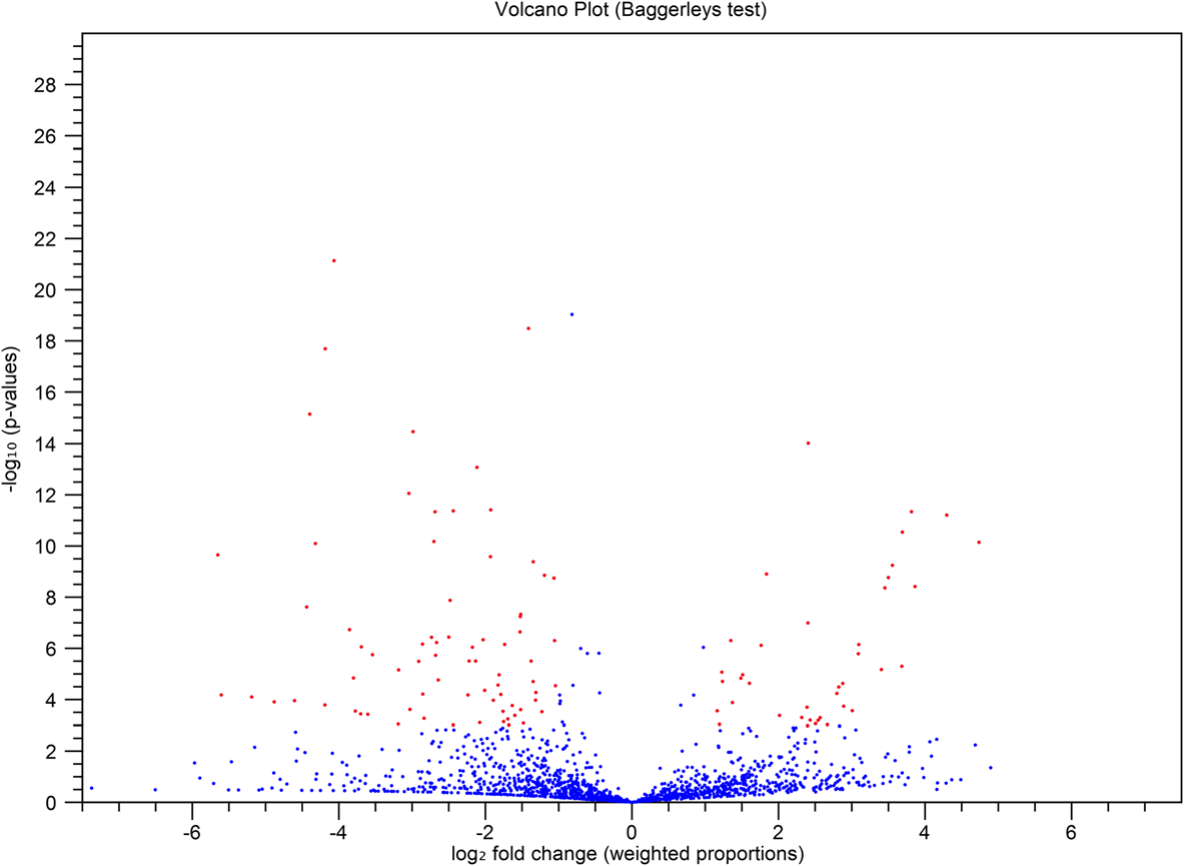
Volcano plot showing overall miRNA expression. The plot was constructed based on the log2 fold change on the x-axis and –log 10 *P*values on the y-axis. The blue and red dots in the plot represent miRNAs. The blue dots at positive values on the x-axis show miRNAs that were not significantly upregulated, whereas red dots at positive values on the x-axis showed miRNAs that were significantly upregulated. The blue dots at negative values on the x-axis showed miRNAs that were not significantly downregulated, whereas the red dots at negative values on the x-axis showed miRNAs that were significantly downregulated

**Fig. 11:**
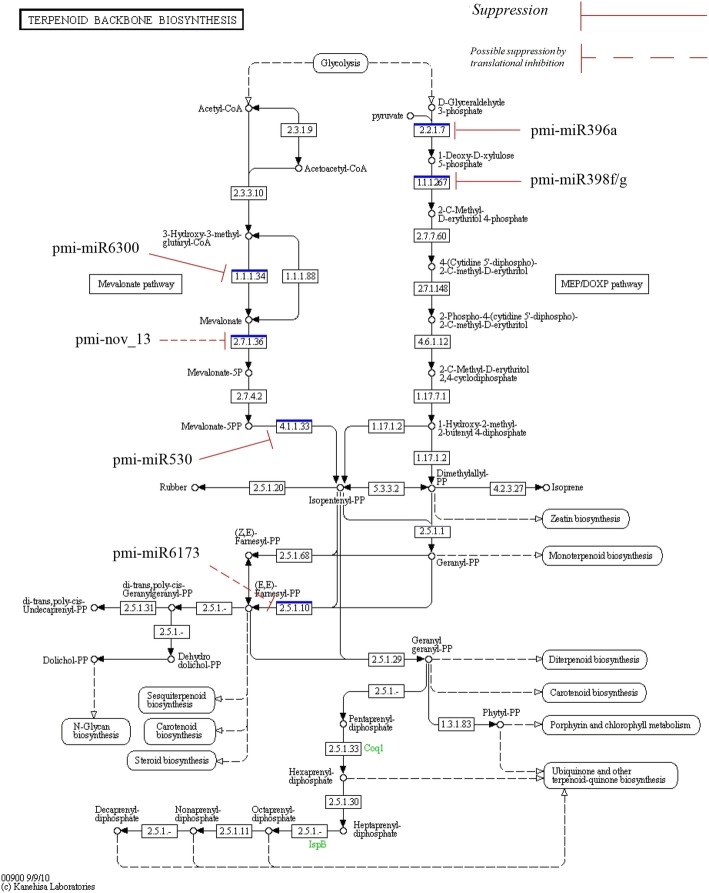
Involvement of miRNAs in the terpenoid pathway in *P. minor* EC 2.2.1.7: 1-deoxy-D-xylulose-5-phosphate synthase; EC 1.1.1.267: 1-deoxy-D-xylulose-5-phosphate reductoisomerase; EC 1.1.1.34: hydroxymethylglutaryl-CoA reductase; EC 2.7.1.36: mevalonate kinase; EC 4.1.1.33: diphosphomevalonate decarboxylase; EC 2.5.1.10: farnesyl diphosphate synthase. The terpenoid biosynthesis backbone pathway was constructed using KEGG software. Suppression symbol (continuous line) indicate the miRNAs had displayed negative relationship against their own target, while dashed suppression symbol indicate the hypothetical effect of miRNAs to inhibit the target via translational inhibition
