# Supplementary material for: Regulation of terpenoid biosynthesis by miRNA in Persicaria minor induced by Fusarium oxysporum
Source: BMC Genomics. 2019 Jul 16;20:586. doi: 10.1186/s12864-019-5954-0 (PMC6636069; doi:10.1186/s12864-019-5954-0)
Supplement: Supplementary file 2 — Measurement of RNA integrity for each sample. An RNA integrity check was carried out prior to small RNA library construction. (PPTX 148 kb) [file 12864_2019_5954_MOESM2_ESM.pptx]

## Slide 1
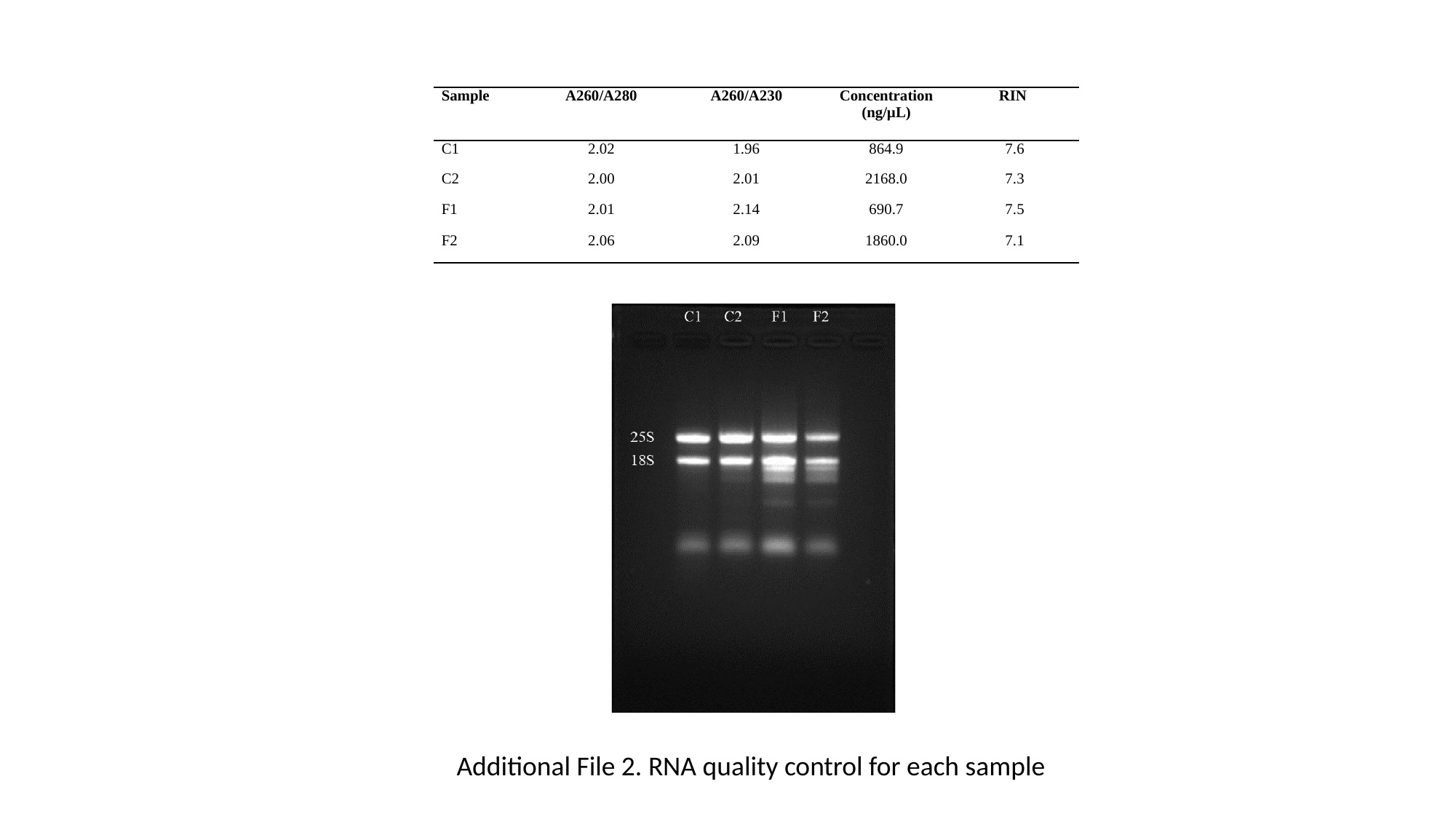

| Sample | A260/A280 | A260/A230 | Concentration (ng/µL) | RIN |
| --- | --- | --- | --- | --- |
| C1 | 2.02 | 1.96 | 864.9 | 7.6 |
| C2 | 2.00 | 2.01 | 2168.0 | 7.3 |
| F1 | 2.01 | 2.14 | 690.7 | 7.5 |
| F2 | 2.06 | 2.09 | 1860.0 | 7.1 |
Additional File 2. RNA quality control for each sample
